# Cyclodepsipeptides: A Rich Source of Biologically Active Compounds for Drug Research

**DOI:** 10.3390/molecules190812368

**Published:** 2014-08-15

**Authors:** Sivatharushan Sivanathan, Jürgen Scherkenbeck

**Affiliations:** Bergische Universität Wuppertal, Fachgruppe C, Organic Chemistry, Gaußstraße 20, Wuppertal 42119, Germany; E-Mail: sivatharushan@googlemail.com

**Keywords:** natural product, cyclodepsipeptide, solid-phase synthesis, biological activity, total synthesis

## Abstract

Faced with the need to find new drugs for all kinds of diseases, science sees that Nature offers numerous classes of compounds showing an impressively high biological potential. Among those are the cyclodepsipeptides, hybrid structures composed of amino and hydroxy acids. In the past decades numerous cyclodepsipeptides have been isolated and their potential as drugs has been studied extensively. For several cyclodepsipeptides total syntheses both in solution and on solid-phase have been established, allowing the production of combinatorial libraries. In addition, the biosynthesis of specific cyclodepsipeptides has been elucidated and used for the chemoenzymatic preparation of nonnatural analogues. This review summarizes the recent literature on cyclic *tetra-* to decadepsipeptides, composed exclusively of α-amino- and α-hydroxy acids.

## Table of Contents

1. Introduction123692. Biosynthesis of Cyclodepsipeptides123703. Cyclotetradepsipeptides123713.1. AM-Toxins123714. Cyclopentadepsipeptides123744.1. Sansalvamide A, N-methylsansalvamide and neo-N-methylsansalvamide123744.2. Alternaramide123764.3. Zygosporamide123765. Cyclohexadepsipeptides123785.1. Enniatins and Beauvericin123785.1.1. Synthesis of Enniatins and Enniatin Derivatives123795.1.2. Biosynthesis of Enniatins and Beauvericin123815.2. Beauvenniatins123845.3. Hirsutellide A123855.4. Kutznerides123865.5. Monamycins123875.6. Himastatin123875.7. Paecilodepsipeptide A and Conoideocrellide A123905.8. Pullularins A-E123915.9. Hirsutatins A and B123926. Cycloheptadepsipeptides123926.1. HUN-7293123927. Cyclooctadepsipeptides123957.1. Bassianolide123957.2. Verticlide123967.3. PF1022A and Emodepside123977.3.1. Syntheses of PF1022A123997.3.2. Synthesis of PF1022A-Analogues via Total Synthesis124017.3.3. PF1022A Analogues by Direct Derivatization of the Natural Product124047.3.4. Biosynthesis of PF1022A124067.3.5. Mode of Action of PF1022A and Emodepside124068. Cyclononadepsipeptides124088.1. BZR-cotoxins I-IV124088.2. Aureobasidins124099. Cyclodecadepsipeptides124109.1. Clavariopsin A and B1241010. Summary12411Conflicts of Interest12411References12412

## 1. Introduction

Cyclodepsipeptides constitute a large family of peptide-related natural products consisting of hydroxy and amino acids linked by amide and ester bonds. The cyclodepsipeptides family can be subdivided into those with an irregular arrangement of ester groups (e.g., kutznerides) and those composed of regularly alternating amide and ester bonds (e.g., enniatins, PF1022A, valinomycine) [1]. Additional classes of cyclodepsipeptides contain β-hydroxy acids (e.g., theopapuamide, marformycins or neamphamide) or β-amino acids (e.g., destruxins) [2]. Cyclodepsipeptides show a broad spectrum of biological activities including antitumor, anthelmintic, insecticidal, antibiotic, antifungal, immunosuppressant, antiinflammatory and antimalarial activities. One plausible explanation for these manifold biological activities is, that the α-hydroxy acids mimic the corresponding α-amino acids. As a consequence depsipeptides are capable like natural peptides, consisting exclusively of ribosomal amino acids, to interact with numerous proteins. In addition, the cyclic nature and frequent *N*-methylation of amino acid residues confer resistance to hydrolyzing enzymes which results in enhanced oral bioavailability. Due to their unique structural and biological properties cyclodepsipeptides have emerged as promising lead structures for applications in crop protection (enniatins) as well as in human (aureobasidine) and veterinary medicine (PF1022). Emodepside^®^, a semisynthetic derivative of the regular cyclooctadepsipeptide PF1022A has been developed as a commercial anthelmintic.

The main source of cyclodepsipeptides are fermentations of various bacteria, actinomycetes and fungi which usually produce mixtures of structurally closely related structures [3,4]. Furthermore, numerous cyclodepsipeptides have been isolated from plants, algae, and cyanobacteria such as the cryptophycins, which are inhibitors of microtubule assembly with impressive *in vivo* activity against solid tumors [5]. In the recent past marine organisms came more and more into the focus of interest. For instance, solanamides A and B, isolated from the marine bacterium *Photobacterium halotolerance*, were found to inhibit virulence gene expression in the serious human pathogen, *Staphylococcus aureus* [6]. Halipeptins A-D, which show strong antimicrobial and antitumor activities, were isolated from the marine sponges *Haliclona* sp. and *Leisosella cf. Arenifibrosa* [7].

Solid-phase synthesis is the method of choice for the synthesis of peptides due to several remarkable advantages compared to solution synthesis as for instance (i) reagent and building block excesses to improve yields; (ii) simplified work-up procedures and (iii) parallel synthesis of peptides on fully automated synthesizers, to name only a few. In addition, the choice of a suitable carrier material such as the Kaiser oxime allows a cyclizative cleavage and reuse of the resin without the need for regeneration. The high-dilution conditions provided by the immense inner surface of solid-phase resins favor intramolecular cyclizations without the need for large solvent quantities [8]. However, the solid-phase synthesis of depsipeptides is by far not that developed as for peptides. The reasons are basically twofold. First, only a limited number of protecting groups for α-hydroxy acids and coupling reagents for ester formation is available. Second, the use of large excesses of hydroxy acids in the coupling reaction is hampered by their high prices and limited availability compared to ribosomal amino acids.

This review focuses on cyclic *tetra-* to decadepsipeptides, composed exclusively of α-amino- and α-hydroxycarboxlic acids. For other classes of depsipeptides the reader is referred to more specific literature [9,10,11,12].

## 2. Biosynthesis of Cyclodepsipeptides

**Figure 1:**
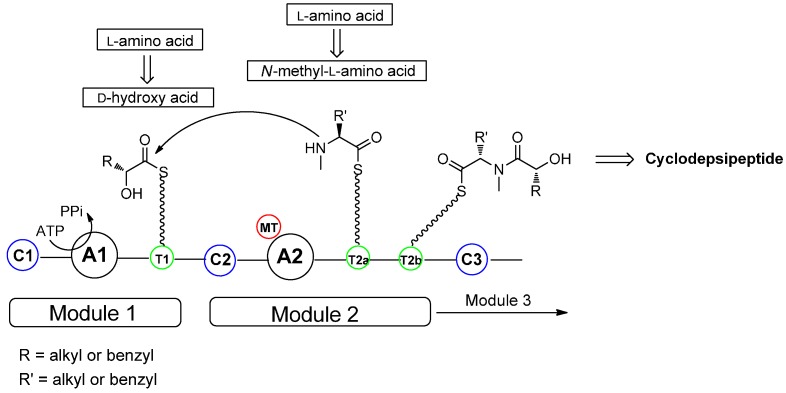
Biosynthesis of NRPs producing cyclodepsipeptides.

In the living cell cyclodepsipeptides are synthesized by giant multi-domain nonribosomal peptide synthetases (NRPs), following the so-called thiol template mechanism which features the domain organization C_1-_A_1-_T_1_-C_2-_A_2-_MT-T_2a-_T_2b_ (Figure 1). The biosynthesis comprises several steps: (1) activation of the *α*-amino acid and the *α*-hydroxy acid as adenylates (A = adenylation domain) using ATP as the co-substrate; (2) capture of those activated substrates as the corresponding thioesters by a phosphopantetheine linker (T = thiolation domain); (3) if necessary, *N*-methylation of amino acids (M domain); (4) condensation of the two substrates, covalently bound to the preceding T domain, forming an enzyme-bound intermediate (C = condensation domain) and (5) final release of the cyclodepsipeptide [13]. While bacterial NRPs use a thioesterase domain (TE) to perform the cyclization, NRPs of fungi terminate with a condensation-like domain (C_T_ domain) [14]. A C-terminal reductase domain (R domain) catalyzes the reductive release of the depsipeptide from the synthetase [15]. Several modifications of the residues such as *N*-methylation, oxidation/reduction (Ox and KR domains), epimerization (E domains) and heterocycle formation (Cy cyclization domains) are performed on specialized domains of those peptide synthetases. In this way dipeptidol monomers are synthesized, which undergo head-to-tail oligmerization forming the desired regular, linear depsipeptide [16]. For instance, bassianolide, a regular cyclooctadepsipeptide, is synthesized as a cyclic tetramer of the dipeptide d-hydroxyisovalerate/*N*-methyl-l-leucine as a linear precursor which is finally cyclized on a C-domain [17].

New, unnatural cyclodepsipeptide analogues can be made available by adding specific, unnatural amino and hydroxy acids either *in vitro* to the multi-domain peptide synthetases or *in vivo* to the producing strain of the microorganism [18].

## 3. Cyclotetradepsipeptides

### 3.1. AM-Toxins

**Figure 2:**
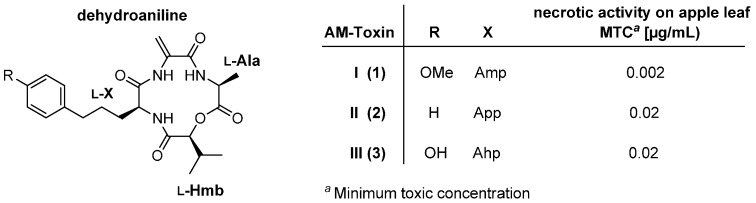
Structures of AM-toxins I-III (**1**–**3**).

AM-toxins (Figure 2), producued by *Alternaria alternate*, are host-specific phytotoxins which cause the spot disease on apple leaves of susceptible cultivars such as Indo and Starking Delicious [19]. AM-toxins consist of l-2-hydroxy-3-methylbutanoic acid (l-Hmb), l-alanine (l-Ala), a (*para*-substituted) l-2-amino-5-phenylpentanoic acid (l-App) and finally the highly reactive Michael-acceptor dehydroalanine which is supposed to be a major cause of the phytotoxic effects of AM-toxins.

l-Amp = l-2-Amino-5-(*p*-methoxyphenyl)pentanoic acid; l-App = l-2-amino-5-phenylpentanoic acid; l-Ahp = l-2-amino-5-(*p*-hydroxyphenyl)pentanoic acid.

**Scheme 1:**
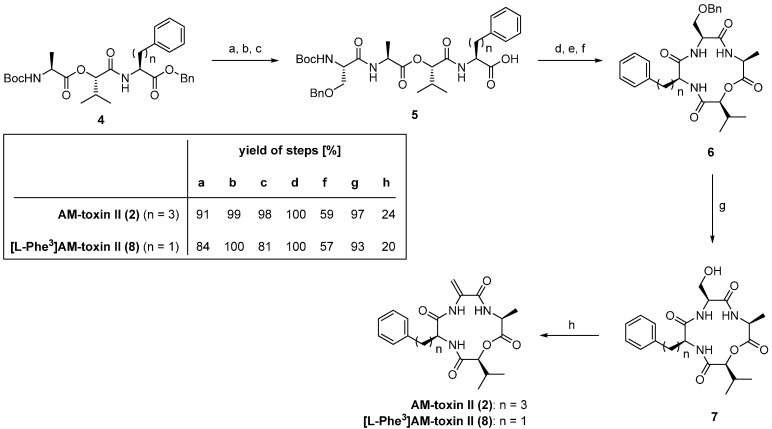
Synthesis of **2** and its derivative **8** described by Waki *et al*.

To date, several total syntheses of AM-toxins I-III (**1**–**3**) were described [20,21]. In 1989 the Waki group presented a total synthesis of AM-toxin II (**2**) and [l-Phe^3^]AM-toxin II (**8**), an analogue of **2**, in which l-2-amino-5-phenylpentanoic acid was replaced for a l-Phe (Scheme 1) [22]. The crucial step of this synthesis was the formation of the dehydroalanine residue which was established in the final step after deprotection using the Miller method (EDCI (1-ethyl-3-(3-dimethylaminopropyl)carbodiimide) and CuCl) for the formal elimination of water. 

**Reagents and conditions:** (a) Pd black, rt, 5 h; (b) 6 M HCl, dioxane, rt, 3 h; (c) Boc-l-Ser(Bzl)-ONSu, Et_3_N, DMF, 0 °C, 1 h; (d) HONSu (*N*-hydroxysuccinimide), EDCI*HCl (1-ethyl-3-(3-dimethylaminopropyl)carbodiimide*hydrochloride), DMF, 4 °C, 24 h; (e) TFA, 0 °C, 30 min; (f) pyridine, DMF, rt, 2 d; (g) Pd black, HCOOH, rt, 2 h; (h) CuCl, EDC*HCl, reflux, 4 d.

**Scheme 2:**
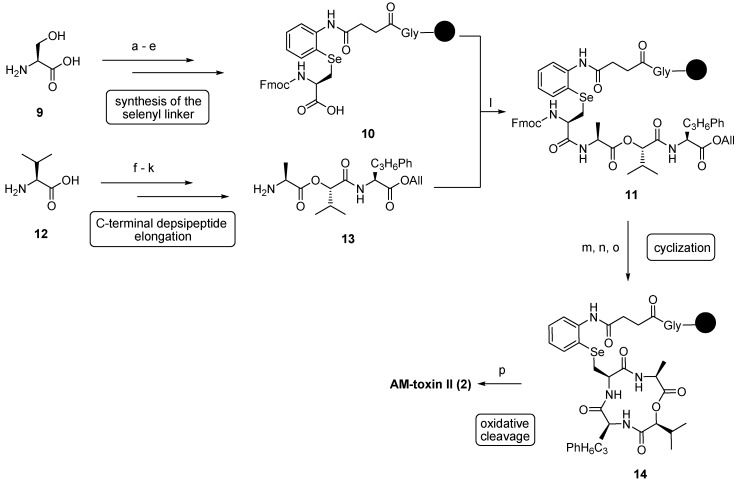
Solid-phase synthesis of AM-toxin II (**2**) by Horikawa.

In 1996 Shirahama *et al.*, established another total synthesis of AM-toxin I (**1**, also called alternariolide) [23]. Here, d-β-phenylselenoalanine was used as precursor for dehydroalanine. Again, the double-bond was established in the final step but now by an oxidative elimination of the phenylselenyl group with anhydrous *tert*-butylhydroperoxide (TBHP) in CH_2_Cl_2_-TFE (5:1) in a yield of 87%. Miyashita and coworkers established the first solid-phase synthesis of AM-toxins on Wang resin [24]. The precursor d-Dap (d-2,3-diaminopropanoic acid) was converted into the dehydro amino acid by a Hofmann degradation with CH_3_I/KHCO_3_ in ethyl acetate. In 2001 Horikawa published an elegant solid-phase synthesis with selenocysteine as a central building block (Scheme 2). The role of selenocysteine was twofold: It was used as precursor for the dehydroalanine and as a linker to the resin. This strategy allowed the formation of the double bond in the final cleavage step [25].

**Reagents and conditions:** (a) *o*-NO_2_-C_6_H_4_-SeCN, Bu_3_P/pyridine, rt, 2 h (83%); (b) Zn, AcOH/THF, rt, 2 h (82%); (c) succinic anhydride, DIPEA/CH_2_Cl_2_, rt, 3 h, (81%); (d) Gly-Wang resin, HATU, DIPEA/DMF, vortex, rt, overnight, (98% in Kaiser test); (e) Pd(PPh_3_)_4_, dimedone/THF, vortex, rt, 3 h; (f) NaNO_2_/AcOH aq., 30 min, (76%); (g) Cs_2_CO_3_, then allyl bromide, rt, overnight, (65%); (h) Boc-Ala, DCC, DMAP/DMF, rt, overnight (73%); (i) cat. Pd(PPh_3_)_4_, piperidine/THF, rt, 2 h, (93%); (j) l-App-*O*All, HATU/DIPEA/DMF, rt, 6 h (88%); (k) 50% TFA/CH_2_Cl_2_, rt, 1 h (99%); (l) HATU, DIPEA/DMF, vortex, rt, overnight; (m) cat. Pd(PPh_3_)_4_, dimedone/THF, vortex, rt, 2 h; (n) 40% piperidine/DMF, vortex, rt, 1 h; (o) FDPP (pentafluorophenyl diphenylphosphinate), DIPEA/DMF, vortex, rt, 2 d; (p) TBHP (*tert*-butyl hydroperoxide)/CH_2_Cl_2_-TFE, vortex, rt, 7 h.

To evaluate the effect of substitution of an amide bond for an ester bond Konzone *et al.*, synthesized a lactam analogue of AM-toxin I containing l-Hmb instead of l-Val [26]. Necrotic activity measurements on apple leaf however, indicated a considerable loss of phytotoxicity (0.1 µg/mL).

## 4. Cyclopentadepsipeptides

### 4.1. Sansalvamide A, N-methylsansalvamide and neo-N-methylsansalvamide

**Figure 3:**
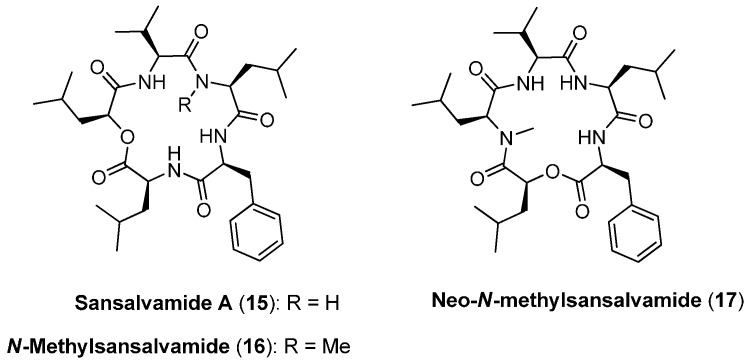
Structures of sansalvamides **15**–**17**.

Sansalvamide (San A, **15**) has been isolated from the organic extracts of the mycelium of a *Fusarium* species collected from the surface of the seagrass *Halodule wrightii* (Figure 3). Sansalvamide A consists of four hydrophobic *α*-amino acids (l-Phe, 2 l-Leu, l-Val) and one hydrophobic *α*-hydroxy acid (l-2-hydroxy-4-methylpentanoic acid) [27]. It exhibits cytotoxic activity against colon and melanoma cancer cell lines showing an *in vitro* IC_50_ value of 9.8 µg/mL towards HCT-116 colon carcinoma. Moreover IC_50_ values of 3.5 and 5.9 µg/mL were measured for the cancer cell-line COLO 205 and melanoma cell-line SK-MEL-2 [28].

A *N*-methylated analogue of sansalvamide A, *N*-methylsansalvamide (**16**), was isolated by Cueto *et al.*, from the extracts of a cultured marine fungus, strain CNL-619, collected in the US Virgin Islands. *N*-methylsansalvamide shows weak *in vitro* cytotoxicity in the NCI human tumor cell line screen (GI_50_ 8.3 µM) [29].

Another analogue of sansalvamide A (**15**), neo-*N*-methylsansalvamide (**17**) was found in extracts from *Fusarium solani* KCCM90040, isolated from infected potatoes in Korea. Neo-*N*-methylsansalvamide (**17**) differs from cyclodepsipeptide **15** only by a different order of the identical residues. Neo-*N*-methylsansalvamide expressed *in vitro* cytotoxic effects on different human cancer cell lines. The EC_50_ values of *neo*-*N*-sansalvamide against the four human cancer cell lines A549, SK-OV-3, SK-MEL-2 and MES-SA were 10.7 ± 0.15, 11.2 ± 1.23, 10.02 ± 0.53, and 14.0 ± 0.74 µM, respectively [30]. 

**Scheme 3:**
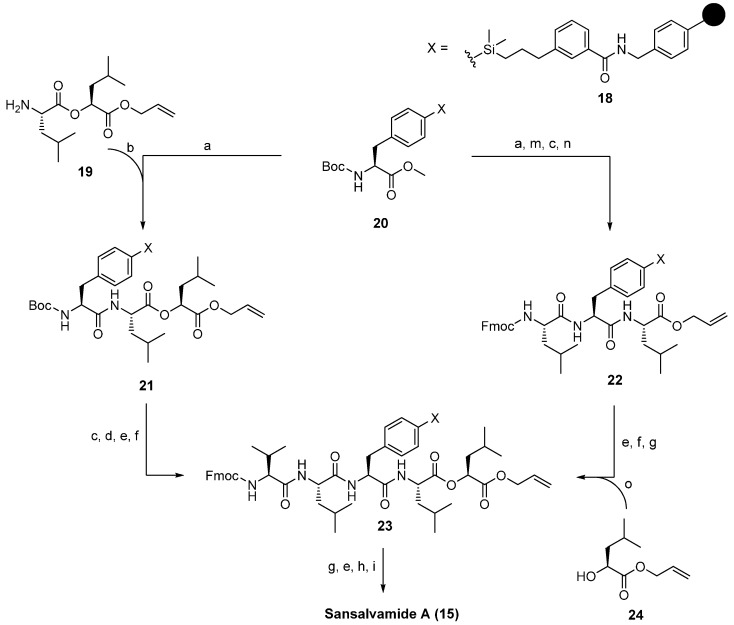
Solid-phase synthesis of **15** by Silverman and coworkers.

A straightforward solid-phase synthesis was established by Silverman in 2000 [31]. The use of an arylsilane-based traceless linker allowed a synthesis in both *N*- and *C*-terminal direction (Scheme 3). This strategy was used to prepare sansalvamide analogue libraries. However, on solid-support only amide bonds were established by connecting a didepsipeptide building-block prepared in solution before. The final macrocyclization after removal of the protecting groups was accomplished with the coupling system HBTU (2-(1H-benzotriazole-1-yl)-1,1,3,3-tetramethyluronium hexafluorophosphate), DIPEA (*N*,*N*-diisopropylethylamine) and NMP (*N*-methyl-2-pyrrolidone). Sansalvamide A (**15**) was obtained in a 10-step total synthesis with an overall yield of 67% and a purity of ˃95%. Remarkably, no high-dilution conditions were needed for the formation of the macrocycle. In addition, Silverman developed an alternative solid-phase strategy in which the ester-bond formation was performed directly on the support. This improved the efficiency of the synthesis considerably and allowed the automated synthesis of sansalvamide analogues. Protolytic cleavage of the product from the arylsilyl traceless linker was accomplished under acidic conditions with 50% TFA/CH_2_Cl_2_.

It is worthwhile to note that the all-amide analogue of sansalvamide A (**15**), san A-amide, was shown to be 10-fold more active (IC_50_ 0.98 µg/mL) in a cell-based cytotoxicity assay against HCT-116 human colon compared to the natural product **15** [32]. In the recent past, mainly the groups of Silverman and McAlpine prepared more than 100 sansalvamide A analogues, both on solid-phase and in solution. Some of those showed excellent chemotherapeutic activities [33,34,35,36,37,38,39].

**Reagents and conditions:** (a) LiOH, THF/H_2_O (7:1), rt, 16 h; (b) HBTU, DIPEA, NMP, 16 h; (c) 2% thioanisole and 50% TFA in CH_2_Cl_2_, 15 min; (d) Fmoc-Leu-OH, HBTU, DIPEA, NMP, 6 h; (e) 20% piperidine in DMF 40 min; (f) Fmoc-Val-OH, HBTU, DIPEA, NMP, 6 h; (g) CHCl_3_/AcOH/NMM, Pd(PPh_3_)_4_, 3 h; (h) HBTU, DIPEA, NMP, 16 h; (i) 2% thioanisole and 50% TFA in CH_2_Cl_2_, 36 h; (j) allyl bromide, K_2_CO_3_, acetone, 48 h (95%); (k) Boc-Leu-OH, DMAP (cat.), DCC, CH_2_Cl_2_, 48 h (63%); (l) 50% TFA in CH_2_Cl_2_, 5 min; (m) O-allyl-Leu, HBTU, DIPEA, NMP, 16 h; (n) Fmoc-Leu-OH, HBTU, DIPEA, NMP, 6 h; (o) MSNT (1-(mesitylene-2-sulfonyl)-3-nitro-1,2,4-triazole), NMI (1-methylimidazole), CH_2_Cl_2_, 16 h.

### 4.2. Alternaramide

**Figure 4:**
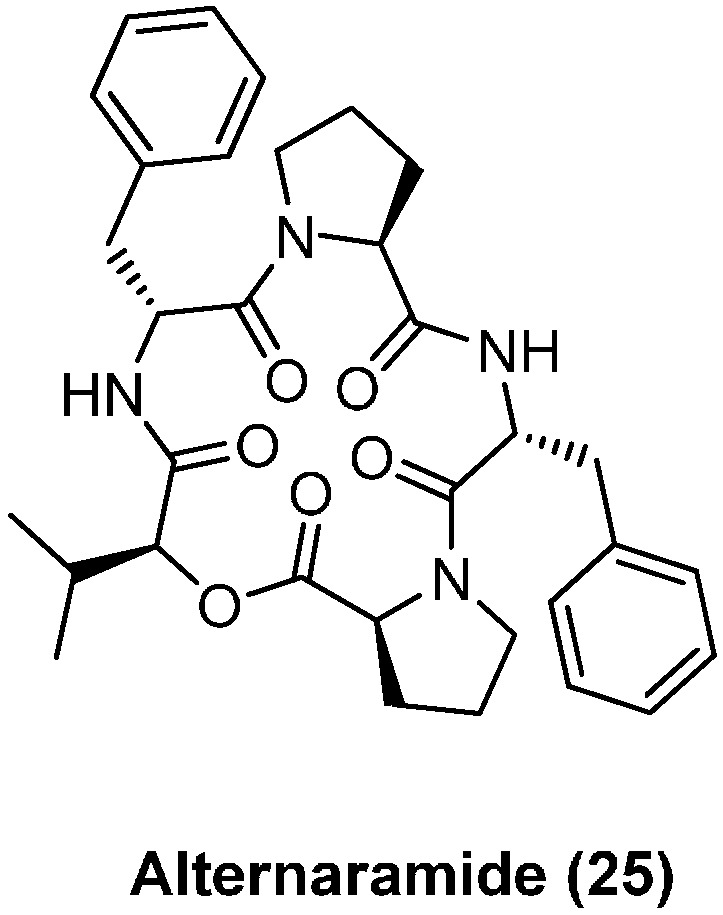
Structure of alternaramide (**25**).

Alternaramide (**25**), composed of two d-Phe, two l-Pro and one l-Hiv (α-l-hydroxyisovaleric acid), was isolated from the fungus *Alternaria* sp. SF-5016 (Figure 4). Weak antibiotic activities against *Bacillus subtilius* and *Staphylococcus aureus* were reported [40]. A single total synthesis has been published to date [41].The residues involved in the macrocycle formation were found to be absolutely critical. A macrolactonization between the residues l-Pro and l-Hiv with excess of PyBOP and DIPEA gave a cyclization yield of only 5%. A macrolactamization between l-Hiv and l-Phe with the same reagent system afforded alternaramide (**25**) in 48% yield.

### 4.3. Zygosporamide

**Figure 5:**
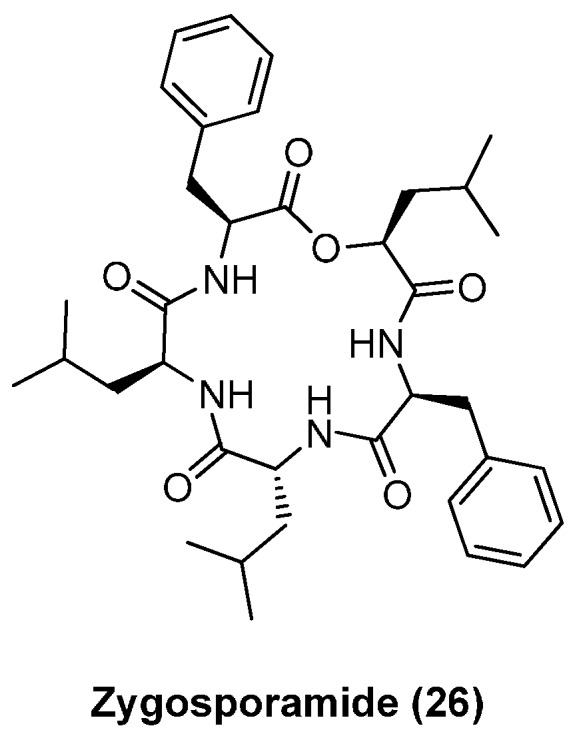
Structure of zygosporamide (**26**).

Zygosporamide (**26**, Figure 5) is produced by the marine-derived fungus, *Zygosporium masonii* and is known as a potent and selective cytotoxic against SF-268 (GI_50_ 6.5 nM) and RXF 393 (GI_50_ < 5.0 nM) cell lines [42].

**Scheme 4:**
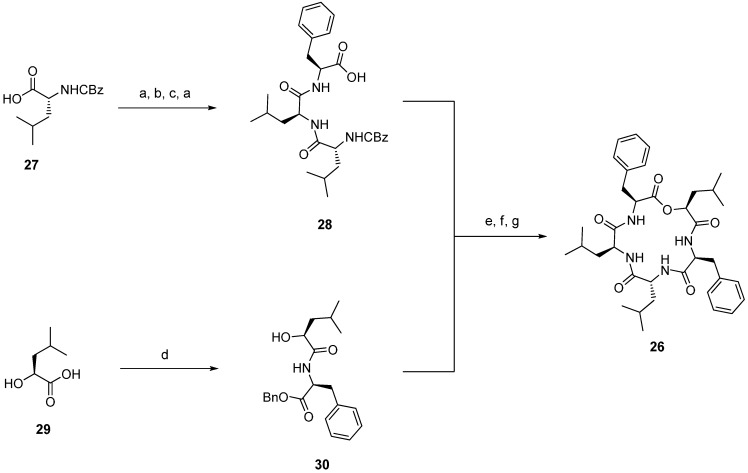
Synthesis of zygosporamide.

In order to establish structure-activity and structure-selectivity relationships, Ma developed a synthesis of zygosporamide (**26**) and prepared a small library of analogues (Scheme 4) [43]. The key step of that synthesis is the coupling of fragments **28** and **30** using the Yamaguchi esterification procedure. The HATU mediated macrocylization under high-dilution conditions gave zygosporamide in a yield of 50%.

**Figure 6:**
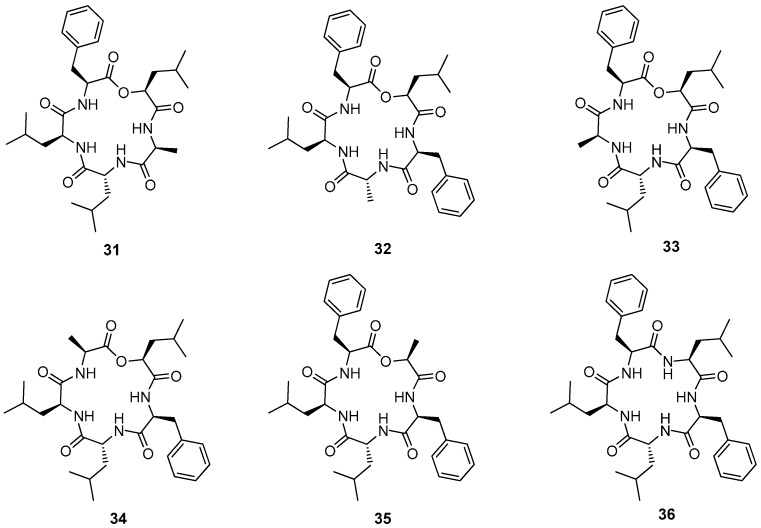
Structures of zygosporamide analogues.

An alanine-scan and replacement of the hydroxy acid by the corresponding amino acid Leu revealed differing structure-activity relationships of cytotoxicity depending on the specific cancer cell line. (Figure 6, Table 1).

**Reagents and conditions:** (a) l-Leu-OMe*HCl, EDC, HOBt, DIPEA, CH_2_Cl_2_ (92%); (b) aq. LiOH, THF, MeOH; (c) l-Phe-OMe*HCl, EDC, HOBt, DIPEA (95%); (d) l-Phe-OBn, BOP, DIPEA, MeCN (90%); (e) TCBC (2,4,6-trichlorobenzoyl chloride, Yamaguchi reagent), DIPEA, DMAP, toluene (80%); (f) Pd/C, H_2_, MeOH; (g) HATU, DIPEA, DMF/CH_2_Cl_2_, (1:4), 0.002 M (50%).

**Table 1:** Biological activities of zygosporamide analogues.

Compound	IC_50_ (µM)				
	SF-268	SF-295	A549	MDA-MB-231	HCT-116
**Zygosporamide (26)**	0.0065	15	7.4	8.5	11.5
**31**	~35	19.7 ± 8.8	~31	7.5 ± 6.3	>50
**32**	10.4 ± 1.5	8.7 ± 0.9	>50	5.0 ± 1.8	2.1 ± 0.4
**33**	>50	>50	>50	>50	>50
**34**	8.8 ± 1.5	~6	>50	2.8 ± 2.1	2.7 ± 0.6
**35**	~31	~32	>50	>50	7.8 ± 6.0
**36**	2.05 ± 2.0	14.4	21.0 ± 5.9	>50	1.9 ± 1.3

## 5. Cyclohexadepsipeptides

### 5.1. Enniatins and Beauvericin

The enniatins and beauvericin (**60**) are known for a long time as fungal mycotoxins mainly from *Fursarium* spp. but also from *Verticillium hemipterigenum* and *Halosarpheia* sp. Additionally, beauvericin was isolated from the hypocrealean entomopathogens *Beuveria bassiana*, *Peacilomyces fumoso-roseus* and *P. tenuipes*.

**Figure 7:**
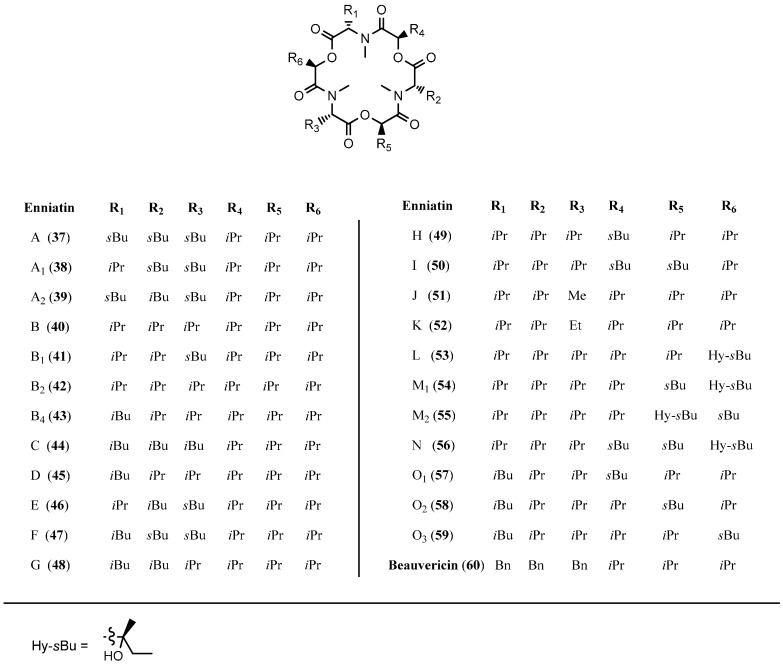
Structures of enniatins **37**–**59** and beauvericin (**60**).

Enniatins and beauvericin (**60**) form a family of regular cyclohexadepsipeptides consisting of three hydroxy acids and three *N*-methyl amino acids in an alternating order. Enniatins were first described over 60 years ago. Up to date 29 different enniatins were isolated and characterized, 18 of those as single compounds, the other ones as mixtures of structurally closely related homologs (Figure 7). *N-*Me-Val, *N-*Me-Leu and *N-*Me-Leu are the common amino acids, hydroxyisovaleric acid (Hiv) is the typical hydroxy acid found in most enniatins [44].

A wide range of biological activities has been reported for enniatins and the related beauvericin (**60**) such as inhibition of the mammalian cholesterol acyl transferase, binding to the γ-aminobutyric acid receptor, activating calcium-sensitive cell apoptose pathways and antiproliferative activity against different human cancer cell lines [45,46]. Recently it was shown that beauvericin also inhibits migration of the metastatic prostate cancer (PC-3M) and breast cancer (MDA-MB-231) cells [47]. In general, due to their ionophoric properties, enniatins and beauvericin (**60)** cause non-specific rat cortical membrane disruption *in vitro* [48].

#### 5.1.1. Synthesis of Enniatins and Enniatin Derivatives

**Scheme 5:**
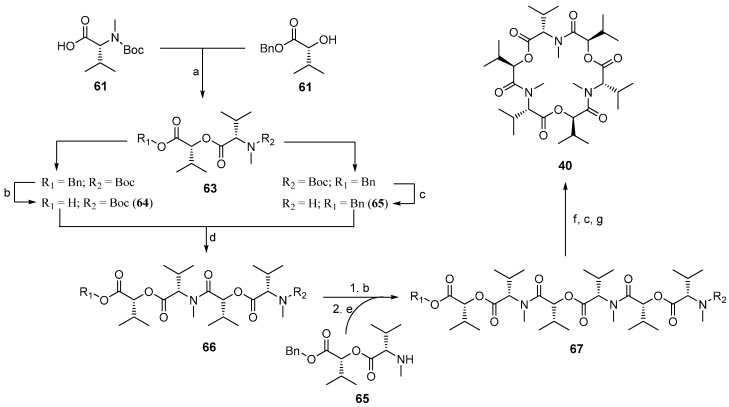
Total synthesis of enniatin B (**40)** by Ley *et al.*

The first synthesis of an enniatin (enniatin B, **40**) was described by Shemyakin in 1963 [49]. In order to establish a flow-synthesis for enniatin **40**, a remarkably optimized synthesis was published by Ley in 2012 (Scheme 5) [50]. The coupling reagents EDCI for ester bond formation and Ghosez’s reagent (1-chloro-*N*,*N*,2-trimethylpropenylamine) for the formation of amide bonds gave excellent yields. In particular, in the critical macrocyclization step under high-dilution conditions Ghosez’s reagent proved superior. The Ley synthesis provided enniatin B in nine steps with an overall yield of 36%.

**Scheme 6:**
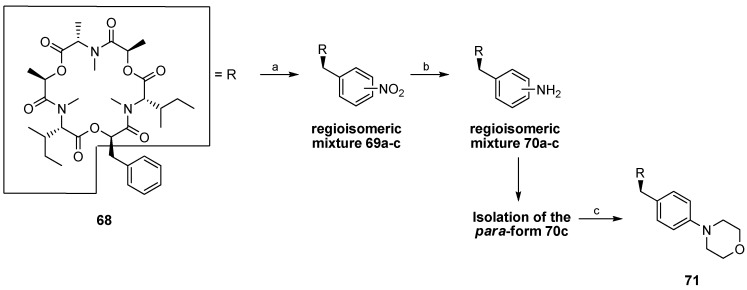
Derivatization of enniatin analogue **68**.

In order to get more information on the potential of enniatins as lead structures for new drugs in the field of crop protection, medicine and animal health numerous analogues were synthesized during the past 20 years. In several cases improved or different biological activities were found. For instance, the replacement of one *N*-methyl-(l)-isoleucine (*N-*Me-Ile) in enniatin A by an *N*-methyl-(l)-alanine (*N-*Me-Ala) caused a 10-fold higher activity against the parasitic nematode *H. contortus* in sheep. Impressive anthelmintic activities were also found for the morpholino-phenyllactic acid containing enniatin derivatives **70a**, **70b**, and **71** (Table 2) which were available by nitration of the phenyl ring, reduction of the nitro-derivative and subsequent reductive amination (Scheme 6) [51].

**Reagents and conditions:** (a) EDCI, DMAP, CH_2_Cl_2_ (82%); (b) H_2_, Pd/C, EtOAc (100%); (c) 4 M HCl/1,4-dioxane (100%); (d) Ghosez's reagent (1-chloro-*N*,*N*,2-trimethyl-1-propenylamine), DIPEA, CH_2_Cl_2_ (91%); (e) 65, Ghosez's reagent, DIPEA, CH_2_Cl_2_, (86%); (f) H_2_, Pd/C, EtOAc (88%); (g) Ghosez's reagent, DIPEA, 0.005 M CH_2_Cl_2_ (56%).

**Table 2:** Anthelmintic activity of enniatin derivatives **68**–**71**.

Enniatin Derivative	Anthelminctic Activity against *H. Contortus*
**68**	0.25 ^a^/0 ^b^
**69c** (*para*)	0.10/1
**70a** (*ortho*)	0.05/3
**70b** (*meta*)	0.05/3
**70c** (*para*)	0.10/1
**71**	0.10/3

(a) Dose in mg test substance kg^−1^ body weight (sheep); (b) 0 = < 50% egg reduction, 1 = 50%–75% egg reduction, 2 = 75%–95% egg reduction, 3 = ˃95% egg reduction.

**Scheme 7:**
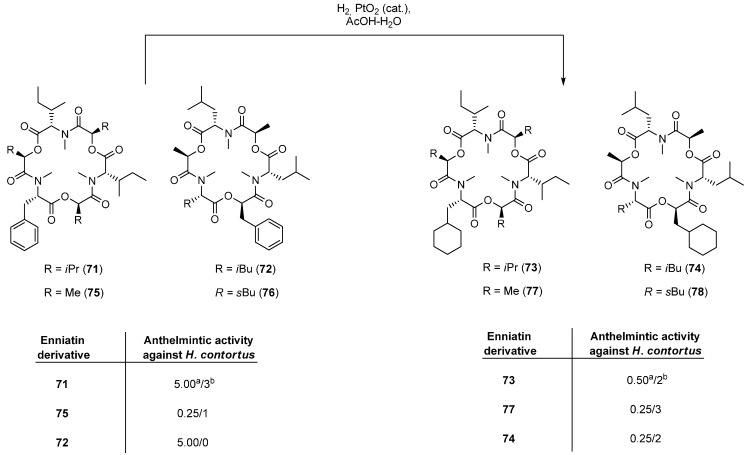
Structures and activities of cyclohexyl-analogues of enniatins.

Based on the observation that the exchange of *N-*Me-Ile by *N-Me*-Phe in enniatin A improves the anthelmintic activity, enniatin derivatives **73**, **74**, **77** and **78** were prepared by hydrogenation (PtO_2_, H_2_, 4–5 bar) of the phenylalanine residue. Even those cyclohexyl-enniatins showed a good to excellent anthelmintic *in vivo* activity in sheep (Scheme 7) [52].

**Reagents and conditions:** (a) fumic nitric acid, 10 °C, 1 h: (b) 20% Pd(OH)_2_, H_2_; (c) 1. 2,5-dihydrofuran, ozone gas, MeOH, CH_2_Cl_2_, −60 °C, 2. NaBH_3_CN, −50 °C, 10 min.

(a) Dose in mg test substance kg^−1^ body weight (sheep); (b) 0 = < 50% egg reduction; 1 = 50%–75% egg reduction; 2 = 75%–95% egg reduction; 3 = > 95% egg reduction.

#### 5.1.2. Biosynthesis of Enniatins and Beauvericin

**Figure 8:**
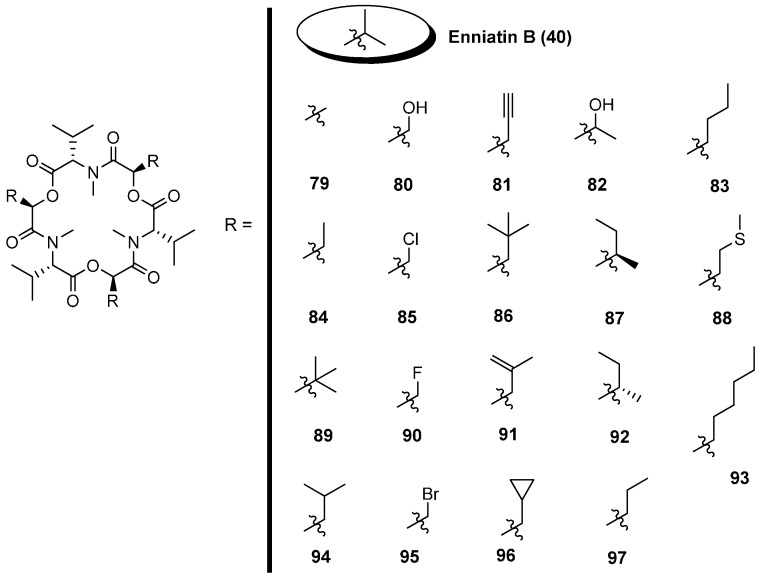
*In vitro* synthesis of hydroxy acid derivatives of **40**.

The first cloned and characterized cyclodepsipeptide synthesase was the enniatin synthetase ESyn, a 347-kDa multienzyme. Compared to other NRPSs, Esyn possesses only two substrate activation modules, EA and EB. While EA is responsible for the activation of α-d-hydroxy acids, EB activates amino acids [53]. The *N*-methylation of the amino acid residues is performed by the N-methyltransferase domain located in module EB. The methyl group is transferred from *S*-adenosylmethionine (AdoMet) to the amino acid. The occurrence of numerous natural enniatins is an evidence of a high tolerance of cyclodepsipeptide synthetases for different amino and hydroxy acids. Zocher and coworkers observed distinct amino acid specificities of two different enniatin producers, *Fusarum lateritium* (enniatin B and B1) and *Fusarium sambucinum* (enniatin A) [46]. Esyn from *Fusarum lateritium* shows a high affinity to l-Val, whereas *Fusarium sambucinum* prefers l-Ileu. Based on earlier studies, in which *N*-methyl-l-valine could be replaced successfully by l-α-aminobutyric acid, l-norvaline and l-leucine [54] additional enniatin analogues have been synthesized recently, following two different strategies, namely small scale *in vitro* syntheses using isolated cyclodepsipeptide synthetases and *in vivo* syntheses by feeding the enniatin producing fungi *Fusarium scripi* and *Fursarium sambucinum* [45]. In particular, the *in vitro* synthesis was intensively studied by Süssmuth in the recent past [55]. Albeit the amino acid substrates for Esyn are restricted to aliphatic, sterically not demanding side chains, several new enniatin analogues were made accessible via this elegant *in vitro* synthesis including *N*-methyl-l-alanine, *N*-methyl-l-serine, *N*-methyl-l-threonine and others. Remarkably, the substrate tolerance of Esyn for hydroxy acids is significantly broader. While the limitation to aliphatic residues still applies, bulky alkyl, halogen or hydroxy functionalized side-chains and even propargyl substituents are accepted. Using this chemoenzymatic approach, Süssmuth succeeded in preparing an enniatin B (**40**) library (Figure 8) containing a set of different *α*-d-hydroxy acid derivatives **79**–**97**. However, the minute amounts of enniatin derivatives available by *in vitro* synthesis prevented thorough studies of biological activities up to now. Additional enniatin derivatives were obtained by simple adding of unusual substrates such as d-2-hydroxybuturic acid, d-lactic acid, l-alanine, l-Abu (l-α-aminobutyric acid) l-serine or l-threonine to the producing *Fusarium* strains [45].

Beauvericin synthetase (BbBEAS) consists of a single polypeptide chain with a molecular mass of about 351 kD. Analogous to enniatin biosynthesis, beauvericin (**60**) is also produced by a thiol template mechanism [56,57]. However, the two depsipeptide synthetases differ in their substrate selectivities. Beauvericin synthetase preferably accepts *N*-methyl-l-phenylalanine and some other aliphatic hydrophobic amino acids. The efficiency of incorporation into the cyclodepsipeptide framework decreases with the length of the side chain: *N*-methyl-l-phenylalanine was easily replaced by *ortho-*, *meta-* and *para*-fluoro substituted phenylalanine derivatives, as well as by *N*-methyl-l-leucine, *N*-methyl-l-norleucine and *N*-methyl-l-isoleucine residues.

**Figure 9:**
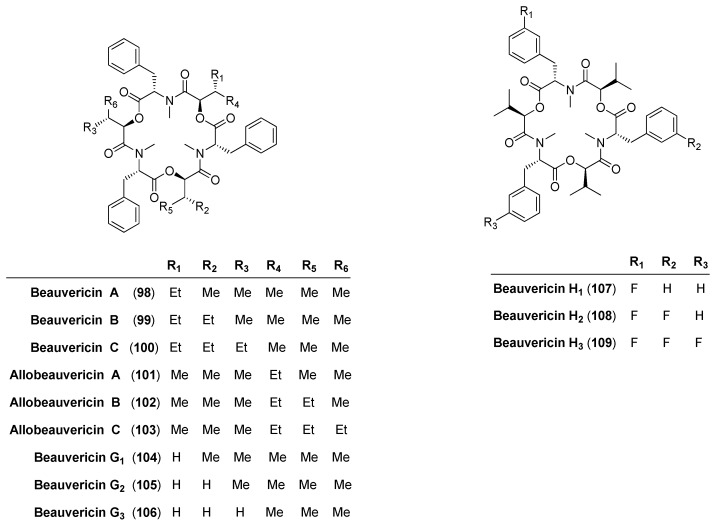
Derivatives **98**–**109** of beauvericin via biosynthesis.

In contrast, *N*-methyl-l-valine a constituent of enniatin B is not accepted. Molnar published six new beauvericin (**104**–**109**) analogues prepared via a precursor-directed biosynthesis with the *Beauveria bassiana* ATCC 7159 strain (Figure 9) [58]. With dl-2-hexabutyric acid (Hbu) and dl-3-fluorophenylalanine as substrates six further beauvericin derivatives **98**–**103** were obtained by Nilanonta *et al.*, from the fungal strain *Peacilomyces tenuipes* BCC 1614 [59].

**Figure 10:**
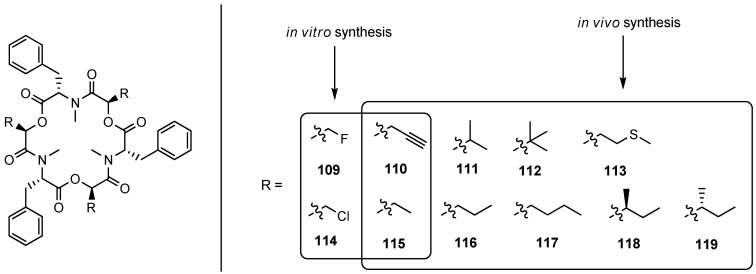
*In vitro* and *in vivo* synthesis of beauvericin analogues **109**–**119**.

In 2012 Süssmuth, for the first time, used recombinant beauvericin synthetase BbBEAS isolated from *E. coli* to generate new beauvericin analogues. In a subsequent whole cell *in vivo* approach the production of beauvericins by mutational biosynthesis using a KIVR knockout strain of *B. bassiana* was studied [60]. Similar to Esyn also BbBEAS shows a broader substrate tolerance for hydroxy acids as exemplified by cyclodepsipeptides **109**–**119** (Figure 10).

### 5.2. Beauvenniatins

**Figure 11:**
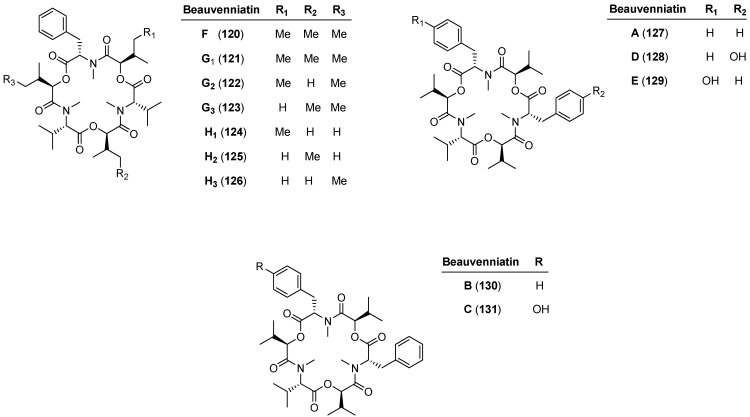
Structures of beauvenniatins **120**–**131**.

A few publications describe hybrid structures between the aliphatic, enniatin-type and aromatic, beauvericin-type cyclohexadepsipeptides, the so-called beauvenniatins (Figure 11, Table 3). Beauvenniatins A–E (**127**–**131**) were isolated from *Acremonium* sp., strain BCC 28424 and beauvenniatins F, G_1_, G_2_, G_3_, H_1_, H_2_, and H_3_ (**120**–**126**) from strain BCC 2629 [61,62].

**Table 3:** Biological activity of beauvenniatins **120**–**131**.

Beauvenniatin	Cytotoxicity (IC_50_)			Antimalaria (IC_50_)	Antituberculosis (IC_50_)
	NCI-H187 ^g^	KB ^h^	Vero ^i^		
**127**	1.0 ^a^	1.3 ^a^	2.2 ^a^	3.0 ^c^	3.13 ^e^
**130**	0.92 ^a^	1.6 ^a^	1.3 ^a^	3.0 ^c^	3.13 ^e^
**131**	6.6 ^a^	14 ^a^	7.0 ^a^	3.4 ^c^	˃50 ^e^
**128**	˃50 ^a^	˃50 ^a^	˃50 ^a^	˃10 ^c^	˃50 ^e^
**129**	7.6 ^a^	9.0 ^a^	8.3 ^a^	2.9 ^c^	25 ^e^
**120**	2.29 ± 1.26 ^b^	1.05 ± 0.05 ^b^	5.5 ^b^	3.8 ± 0.1 ^d^	1.07 ^f^
**121–123**	1.23 ± 0.49 ^b^	1.06 ± 0.06 ^b^	4.1 ^b^	3.9 ± 0.4 ^d^	2.18 ^f^
**124–126**	1.45 ± 0.38 ^b^	1.15 ± 0.11 ^b^	1.9 ^b^	3.6 ± 0.9 ^d^	4.45 ^f^

(a) IC_50_ (µg/mL); (b) IC_50_ (µM); (c) antimalarial activity against *P. falciparium* K1, showed in IC_50_ (µg/mL); (d) antimalarial activity against *P. falciparium* K1, showed in IC_50_ (µM); (e) antituberculosis activity against *M. tuberculosis* H37Ra, showed in IC_50_ (µg/mL); (f) antituberculosis activity against *M. tuberculosis* H37Ra, showed in IC_50_ (µg/mL); (g) human small-cell lung cancer; (h) oral human epidermoid carcinoma; (i) African green monkey kidney fibroblasts.

### 5.3. Hirsutellide A

**Figure 12:**
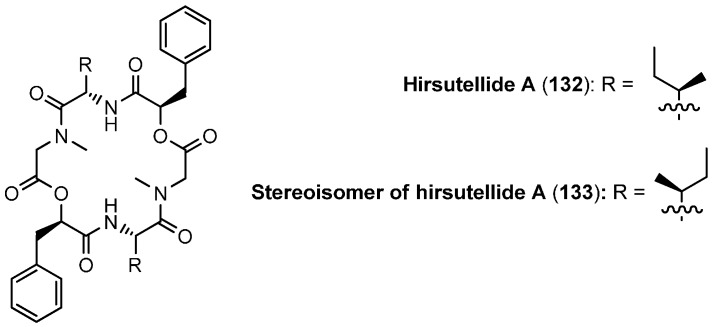
Structure of hirsutellide A and its stereoisomer.

Hirsutellide A (**132**, Figure 12), another 18-membered cyclodepsipeptide, was isolated from the cell extracts of *Hirsutella kobayasii* BCC 1660 [63]. It exhibits antimycobacterial activity (MIC 6–12 µg/mL; activity against *Mycobacterium tuberculosis*) and also weak *in vitro* antimalarial activity (IC_50_ 2.8 µg/mL; activity against *Plasmodium falciparum*). However no cytotoxic effects were found against Vero cells at 50 µg/mL . A conventional solution synthesis, using standard reagents for ester (DCC, (*N*,*N*'-dicyclohexylcarbodiimide) and amide bond (HATU) formation was developed by Xu [64,65]. In fact, the macrocylization reaction turned out problematic. BOP-Cl (*bis-*(2-oxo-3-oxazolidinyl)-phosphinic chloride) afforded only a low yield of 15% of the cyclic depsipeptide, FDDP a slightly improved yield of 22%.

A drastically improved cyclization yield (61%) was obtained for the stereoisomer **133** of hirustellide A with AgBF_4_ as cyclization reagent [66]. The authors suggest that the silver ion acts as a template, to which the carbonyl and the oxygen or amino groups coordinate. This arrangement in close spatial neighborhood facilitates then the difficult cyclization.

### 5.4. Kutznerides

**Figure 13:**
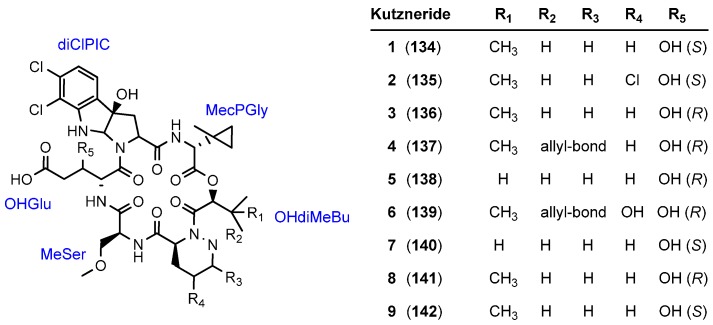
Structure of kutznerides **134**–**142**.

In 2006 Pohanka and Vasiliauskas isolated a novel class of cyclohexadepsipeptides from the actinomycete *Kutzneria* sp. 744. The kutznerides **134**–**142** inhibit the growth of the pathogenic fungi *Pythium undalatum*, *Ceratobasidium bicorne* and *Fusarium avenaceum* and thus illustrate attractive lead structures for novel antifungals (Figure 13) [67,68]. These compounds **134**–**142** represent remarkable structures as they do not contain a single ribosomal amino acid. With respect to the tricyclic tryptophane analogue, the kutznerides pose a formidable challenge for a total synthesis.

**MecPGly** = 2-(1-methylcyclopropyl)-d-glycine, **OHdiMeBu** = (*S*)-2-hydroxy-3,3-dimethylbutanoic acid, **MeSer** = *O*-methyl-l-serine, **OHGlu** = 3-hydroxy-d-glutamic acid, **diCPlC** = (2*S*,3a*R*,8a*S*)-6,7-dichloro-3a-hydroxypyrrolidino[2,3-b]indole-2-carboxylic acid.

The kutznerides were tested against diverse pathogens shown in Table 4. The most potent compounds were the trichloro kutzernerides 2 (**135**) and 8 (**141**), with promising MIC values against *Erwina carotovora* and *Staphylococcus aureus*. In 2007 Walsh identified the biosynthesis gene cluster comprising six nonribosomal peptide synthetase (NRPS) modules distributed over three proteins and a variety of tailoring enzymes including halogenases [69]. The study provides an interesting insight into the biochemical transformations involved in the synthesis of the unique residues of the kutznerides.

**Table 4:** MIC values of kutznerides against various pathogens.

Kutzneride	MIC values (µM) of Pathogens		
	*Drechslera sorokiniana* (agricultural fungus)	*Erwina carotovora* (agricultural bacterium)	*Staphylococcus aureus* (human bacterium)
**1 (134)**	230	60	12
**2 (135)**	110	6	6
**3 (136)**	260	12	9
**4 (137)**	˃590 ^a^	120	140
**5 (138)**	˃240 ^a^	120	120
**6 (139)**	˃400 ^a^	˃230 ^a^	˃230 ^a^
**7 (140)**	˃420 ^a^	180	120
**8 (141)**	230	6	6
**9 (142)**	˃230	230	60

(a) No inhibition at the indicated concentration.

### 5.5. Monamycins

**Figure 14:**
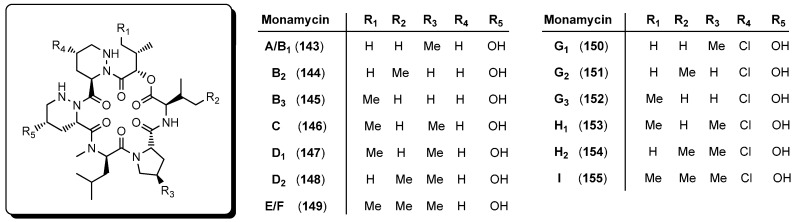
Structures of different monamycins **143**–**155**.

The monamycins **143**–**155** were isolated from *Streptomyces jamaicensis* more than fifty years ago (Figure 14) [70]. In 1970 Hall described potassium, caesium and rubidium complexes of the monamycins **143**–**155** and attributed their antibacterial effects against *Staphylococcus aureus* (IC_50_ 0.5–2.0 µg/mL) to their ionophoric properties causing lysis of the bacterial cell [71]. This class of cyclodepsipeptides attracted much interest when pronounced antibiotic effects against resistant strains of Gram-positive bacteria were found [72]. Bromomonamycin analogues of chloromonamycins G_1_, G_2_, G_3_, H_1_, H_2_, and I were made available by cultivating the fungus in a chloride-free medium to which NaBr was added as halogen source [73]. Unfortunately, the bromomonamycins showed only a reduced antibacterial activity. The monamycins contain the unique building block (3*S*,5*S*)-5-hydroxypiperazic acid which was first synthesized by Hale via a chiral-pool approach starting from d-mannitol [72].

### 5.6. Himastatin

**Figure 15:**
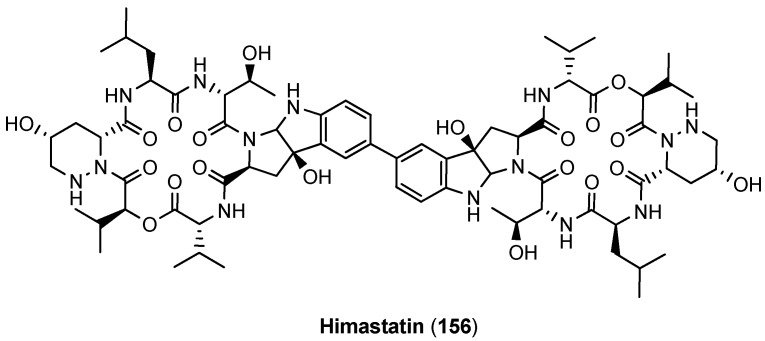
Structure of himastatin (**156**).

Himastatin (**156**) is a highly interesting cyclodepsipeptide due to its strong antimicrobial activity in particular against Gram-positive bacteria and its unique symmetric structure (Figure 15, Table 5) [74]. Himastatin has been found in a strain of *Strepthomyces hygroscopicus* (ATCC 53653) collected in the state of Himachal Pradesh in India. Himastatin represents a dimeric cyclohexadepsipeptide consisting of two tricyclic tryptophan derivatives that are connected by a biphenyl bridge. With (3*R*,5*R*)-5-hydroxypiperazic acid a second unusual building is incorporated in the cyclodepsipeptide framework [75,76,77].

**Table 5:** Antimicrobial activities of **156** [71].

Organism	MIC (µg/mL)	Organism	MIC (µg/mL)
*E. faecalis* A20688	0.5	*E. coli* A20697	˃500
*E. faecalis* A25707	0.5	*E. coli* A9751	2
*E. faecalis* A25707	0.25	*Klebsiella pneumoniae* A9664	˃500
*S.aureus* A9537	0.5	*K. pneumonia* A20468	˃500
*S. aures* A20698	1	*Proteus vulgaris* A21559	˃500
*S. aureus* A24407	1	*Pseudonomas aeruginosa* A9843	˃500
*Bacillus subtilis* A9506-A	2	*P. aeruginosa* A20235)	˃500
*Escherichia coli* A15119	˃500	*P. aeruginosa* A20235	˃500

**Figure 16:**
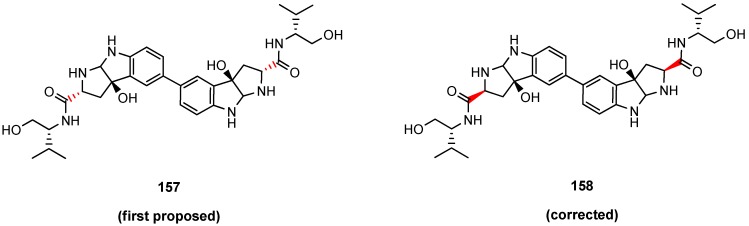
Proposed (**157**) and proven (**158**) structure of the central himastatin degradation product.

The structure of himastatin (**156**) was mainly deduced by degradation experiments. Based on NMR studies Bristol Myers scientists attributed a *trans*-arrangement of the carboxamide and the OH-group in the tryptophan-biphenyl cleavage product (Figure 16) [78]. However, in a seminal total synthesis of himastatin Danishefsky proved the *trans*-arrangement to be wrong [79].

**Scheme 8:**
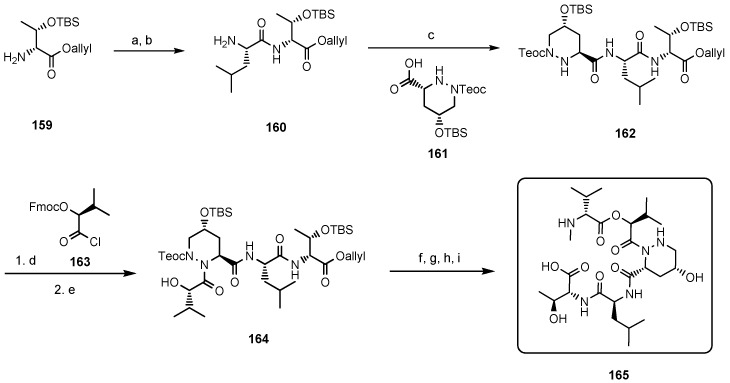
Total synthesis of himastatin: fragment **165**.

**Scheme 9:**
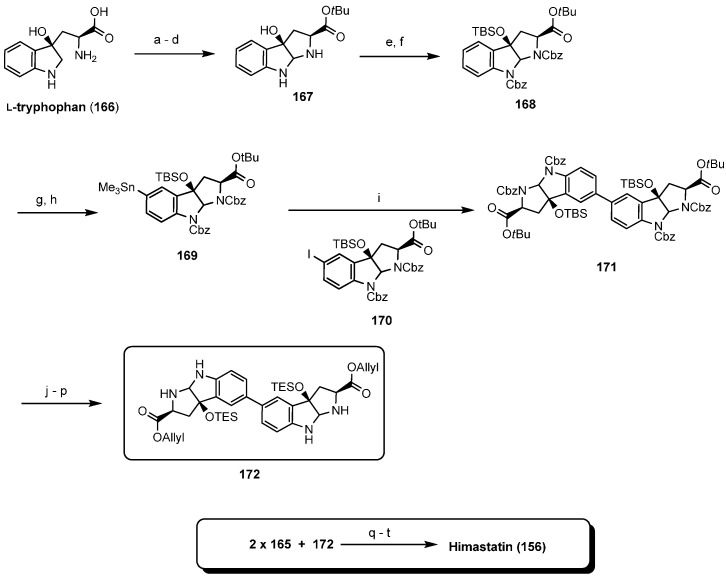
Total synthesis of himastatin **156**: final steps.

In a second total synthesis Danishefsky obtained the correct himastatin stereoisomer which was identical with natural himastatin in all spectroscopic properties [80]. This elegant synthesis was based on two separate fragments, a pentadepsipeptide acid (**165**, Scheme 8) and a pyrroloindoline dimer (**171**, Scheme 9) which were coupled only in the final stage, establishing the dimeric structure of himastatin in a one-pot reaction.

**Reagents and conditions:** (a) Fmoc-l-Leu, EDCl, DMAP, CH_2_Cl_2_; (b) piperidine, CH_3_CN, 76% (over 2 steps; (c) **161**, HATU, HOAt, collidine, CH_2_Cl_2_, 95%; (d) **163**, collidine, CH_2_Cl_2_; (e) piperidine, CH_3_CN, 96% (over 2 steps); (f) Troc-d-valine, IPCC, Et_3_N, DMAP, CH_2_Cl_2_, (g) ZnCl_2_, CH_3_NO_2_; (h) TBSOTf, 2,6-lutidine, CH_2_Cl_2_; (i) [Pd(PPh)_3_]_4_, PhSiH_3_, THF, 72% (over 3 steps).

**Reagents and conditions:** (a) TrCl (trityl chloride), TEA, THF; (b) *tert*-butyl isourea, CH_2_Cl_2_, rt, 76% (over 2 steps); (c) DMDO, CH_2_Cl_2_, −78 °C; (d) HOAc, MeOH, CH_2_Cl_2_, rt, 76% (over 2 steps); (e) CbzCl, py, CH_2_Cl_2_; (f) TBSCl, DBU, DMF, rt (61%); (g) ICl, 2,6-di-*tert*-butylpyridine, CH_2_Cl_2_ (73%); (h) Me_6_Sn_2_, [Pd(PPh_3_)_4_], THF, 60 °C (87 °C); (i) **170**, [Pd_2_dba_3_], Ph_3_As, DMF, 45 °C (83%); (j) TBAF, THF; (k) TMESCl, DBU, DMF (95%); (l) Pd/C, H_2_, EtOAc; (m)Fmoc-HOSu, py, CH_2_Cl_2_ (96%); (n) TESOTf, lutidine, CH_2_Cl_2_, 0 °C to rt; (o) allyl alcohol, DBAD, PH_3_P, THF (81%), (p) piperidine, CH_3_CN (74%); (q) HATU, HOAt, collidine, CH_2_Cl_2_, −10 °C to rt (60%); (r) 1. [Pd(PPh_3_)_4_], PhSiH_3_, THF; 2. Pb/Cd, NH_4_OAc, THF (56%); (s) HOAt, HATU, DIPEA, DMF; (t) TBAF, THF, HOAc (35%).

**Figure 17:**
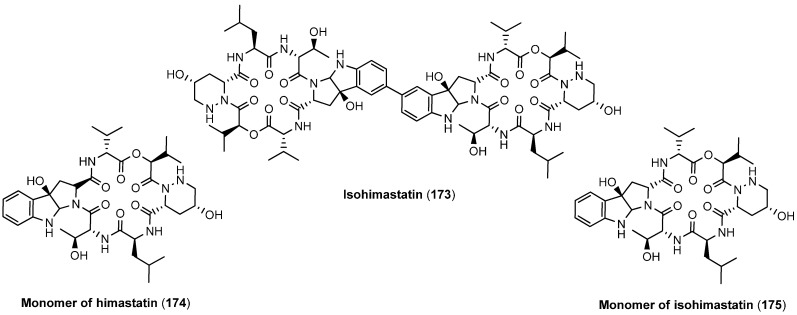
Analogues of himastatin.

Isohimastatin (**173**), originally proposed to be himastatin, the monomers of himastatin and isohimastatins (**174** and **175**), obtained in the course of the total synthesis (Figure 17), were also tested against various Gram-positive bacteria but were found all inactive.

Inspired by its unique structure, Ju explored the biosynthesis of himastatin (**156)** [81]. Himastatin is biosynthesized by a nonribosomal peptide synthetase (NRP) assembly line, that requires prior- and post-oxidative modifications for the formation of the 3-hydroxy-d-Pip residue, the oxygenation of l-Trp and the subsequent symmetrical biaryl coupling. Ju found three cytochrome P450 enzymes responsible for the complete biosynthesis of himastatin: (i) HmtT transforms l-Trp to the tricyclic pyrroloindole unit; (ii) HmtN regio- and stereoselectively hydroxylates d-Pip; and (iii) HmtS catalyses the biaryl coupling.

### 5.7. Paecilodepsipeptide A and Conoideocrellide A

**Figure 18:**
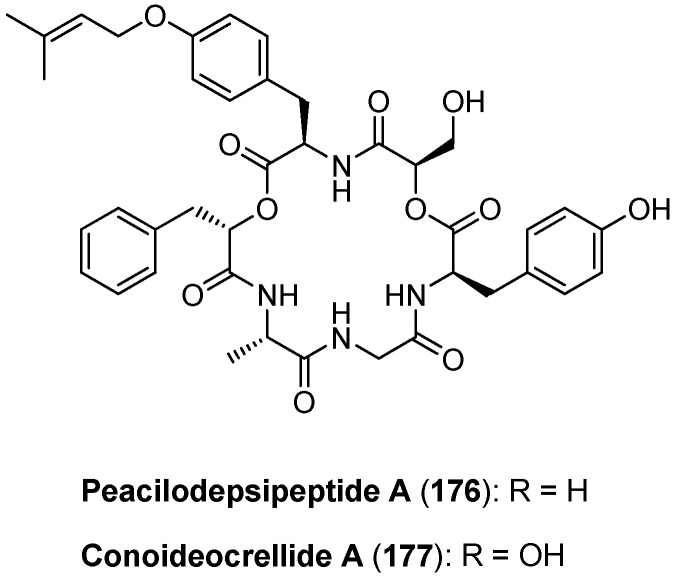
Structure of paecilodepsipeptide A and conoideocrellide A.

Very recently, the cyclohexadepsipeptide conoideocrellide A (**177**) was isolated from *Conoideocrella tenuis* BCC 18627, an insect pathogenic fungus together with its linear precursors conoideocrellides B-D (Figure 18) [82]. A structurally closely related cyclodepsipeptide, paecilodepsipeptide A (**176**), was isolated from *Paecilomyces cinnamomeus* BCC 9616, another insect pathogenic fungus. Paecilodepsipeptide A has activity against the malarial parasite *Plasmodium falciparum* K1 with an IC_50_ of 4.9 µM, and, furthermore, it shows cytotoxicity against two cancer cell lines, KB (IC_50_ = 5.9 µM) and BC (IC_50_ = 6.6 µM). Interestingly, only the cyclic depsipeptide showed biological activity, but not the linear precursors [83].

**Scheme 10:**
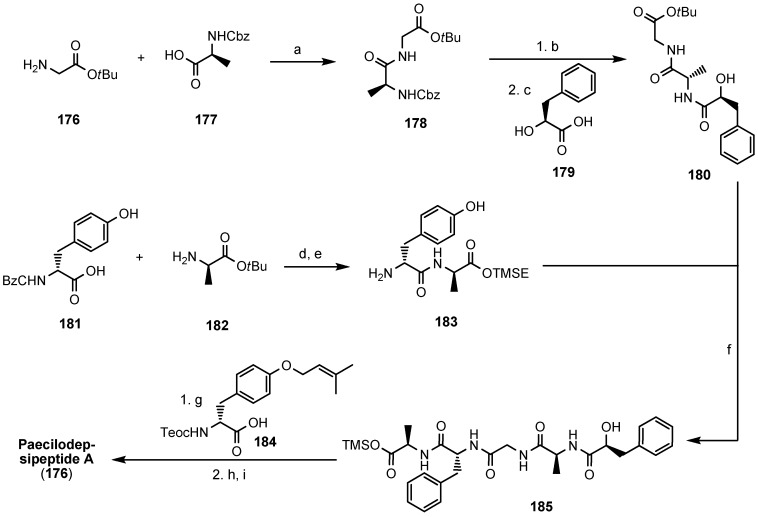
Total synthesis of paecilodepsipeptide A 176.

In 2008 Yang and coworkers detailed a total synthesis of paecilodepsipeptide A (**176**) in solution (Scheme 10). The highest yield (72%) in the critical macrocylization reaction was obtained with HATU as coupling reagent under high-dilution conditions [84].

**Reagents and conditions:** (a) EDCl/HOBt/Et_3_N/CH_2_Cl_2_, 89%; (b) H_2_, Pd/C; (c) **179** EDCl/HOBt/Et_3_N/CH_2_Cl_2_, 86%; (d) EDCl/HOBt/Et_3_N/CH_2_Cl_2_, 90%; (e) H_2_, Pd/C MeOH; (f) EDCl/HOBt/Et_3_N/CH_2_Cl_2_, 76%; (g) **184**, EDCl/HOBt/Et_3_N/CH_2_Cl_2_, 75%; (h) TBAF/THF; (i) HATU/DIPEA/DM, 72%.

### 5.8. Pullularins A-E

**Figure 19:**
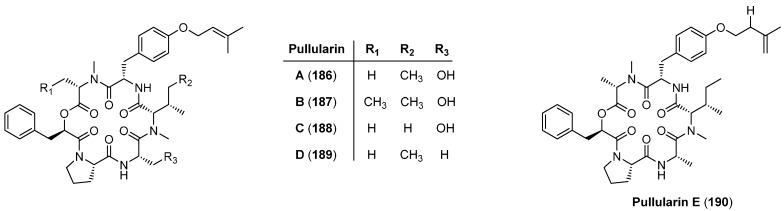
Structures of pullularins A-E (**186**–**190**).

From the fungus *Pullularia* sp*.* BCC 8613 four cyclohexadepsipeptides, pullularins A–D (**186**–**189**) were isolated and characterized by Isaka in 2007 [85]. Pullularin A (**186**) (Figure 19) shows antimalarial, antiviral (herpes) and antitubercular activity. All pullularines share an unusual *O*-prenyl-l-tyrosine residue and a single ester bond in the cycle. In 2012 an additional cyclodepsipeptide, pullularin E (**190**) was published [86]. Pullularin E (**190**) is characterized by an *O*-isopentenyl substituted tyrosine instead of a *O*-dimethylallyl residue as found in the pullularins A–D (**186**–**189**).

### 5.9. Hirsutatins A and B

**Figure 20:**
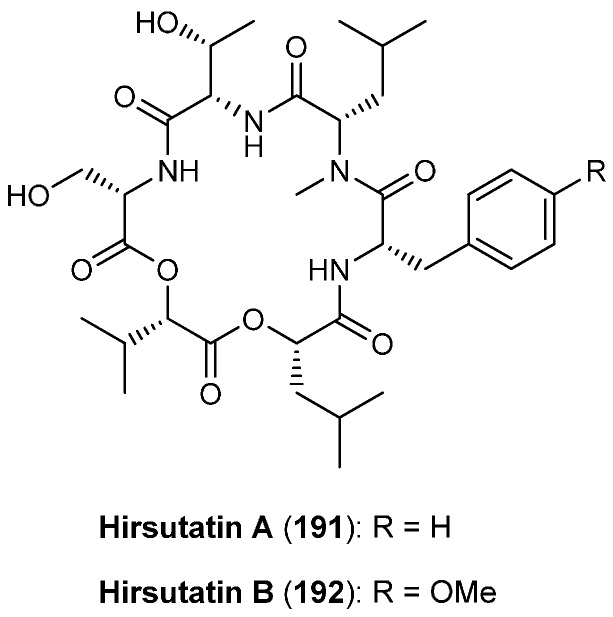
Structure of hirsutatin A (**191**) and B (**192**).

Hirsutatins A (**191**) and B (**192**, Figure 20) as well as the hirsutellones A-E were isolated from the insect pathogenic fungus *Hirsutella nivea* BCC 2594 [87,88]. While hirsutatin B **192** shows antimalarial activity against *Plasmodium falciparum* K1 (IC_50_ 5.8 µg/mL) **191** was found inactive even at a concentration of 20 µg/mL. In contrast to the enniatins, in which the amino acids usually have the l- and the hydroxy acids the d-configuration, the hirsutatine building blocks all have the l-configuration.

## 6. Cycloheptadepsipeptides

### 6.1. HUN-7293

**Figure 21:**
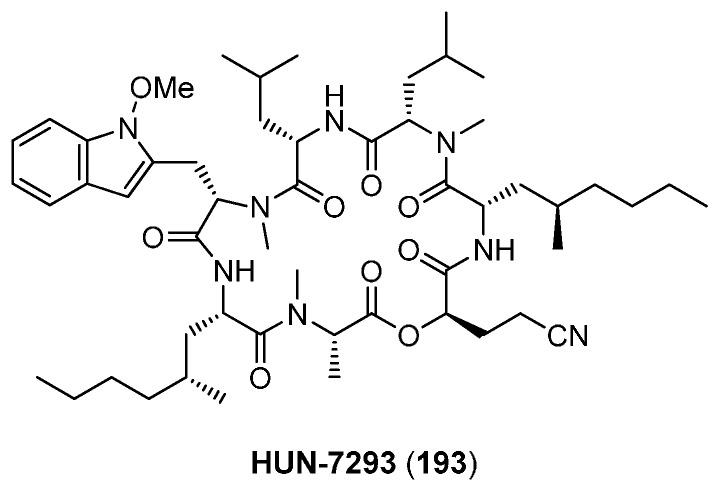
Structure of HUN-7293 (**193**).

Isolated first in 1992 from a fungal broth heptadepsipeptide HUN-7293 (**193**, Figure 21) has emerged as a potent lead structure for the treatment of autoimmune diseases and inflammatory disorders [89]. It acts as an inhibitor of inducible cell adhesion molecule expression (VCAM-1: IC_50_ 1.0 nM, ICAM-1: IC_50_ 24.0 nM, E-selectin: IC_50_ 24.0 nM). Structurally HUN-7293 consists of six l-amino acids, four of which nonribosomal, and one d-*α*-hydroxy acid.

**Scheme 11:**
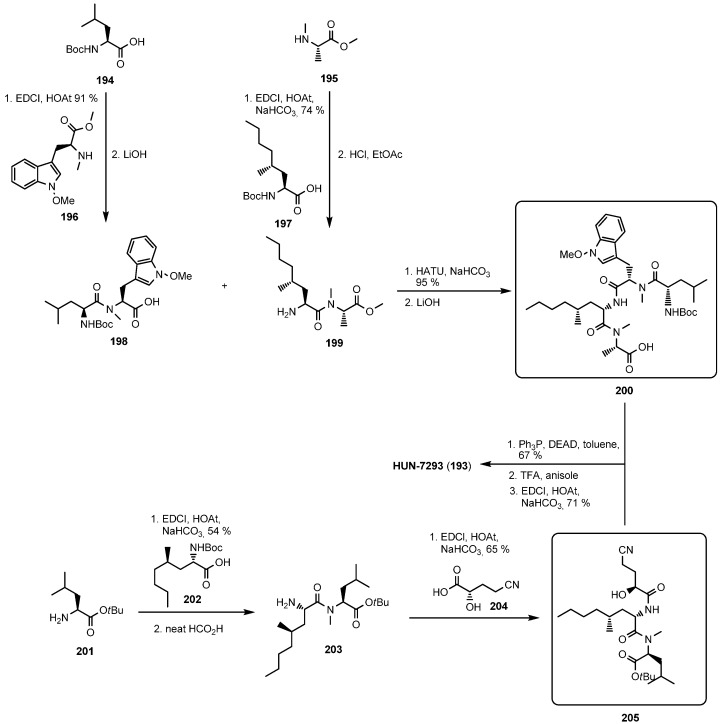
Synthesis of HUN-7293 (**193**).

The first total synthesis of HUN-7293 (**193**) was described by Boger in 1999 [90]. Two strategies were envisaged (Scheme 11) for the critical macrocyclization. In the first route, a linear heptadepsipeptide was prepared, using a modified Mitsonobu reaction to form the ester bond in the final step. After simultaneous removal of the protecting groups under acidic conditions a macrolactamization with EDCI-HOAt afforded the desired product in a yield of 71%. The second route, a macrolactonization, however did not give any cyclodepsipeptide HUN-7293 neither with a Mitsunobu reaction nor with the Steglich esterification method.

**Scheme 12:**
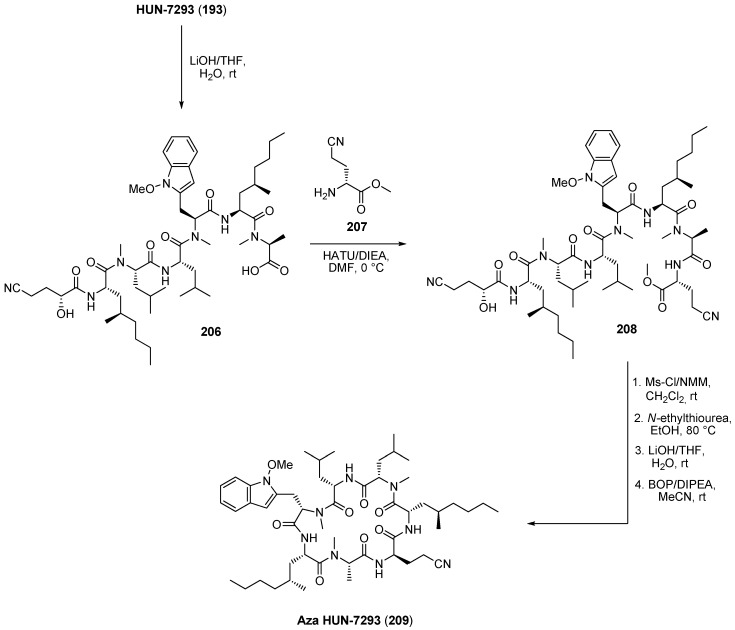
Synthesis of aza-Hun-7293 (**209**) by modification of the natural product.

With an efficient total synthesis at hand, an aza-analogue of HUN-7293 (aza HUN-7293, **209**) was prepared [91]. In contrast to the macrocyclization conditions used for the total synthesis of the natural product, the coupling system BOP ((benzotriazol-1-yloxy)tris(dimethylamino)phosphonium hexa-fluoro-phosphate)/DMAP was shown to be superior because epimerization was significantly reduced. An alternative method for preparing aza-HUN-7293 by a straightforward modification of the natural product was published by Schreiner and coworkers [92]. The authors successfully described the synthesis of aza-HUN-7293 (**209**) by a ring opening reaction and subsequent substitution of the d-hydroxy acid by the corresponding d-amino acid (Scheme 12).

**Figure 22:**
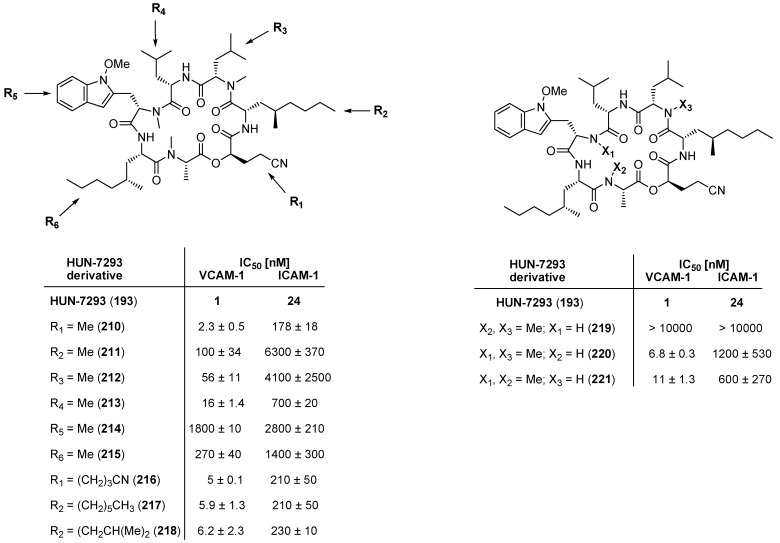
HUN-7293 analogues and their biological activities.

In order to establish structure-activity relationships Boger used solution-phase parallel synthesis to prepare HUN-7293 analogues, including an alanine and a *N*-methyl deletion scan [90]. However, to varying degrees all analogues showed reduced activities against VCAM-1 and ICAM-1 (Figure 22).

## 7. Cyclooctadepsipeptides

### 7.1. Bassianolide

**Figure 23:**
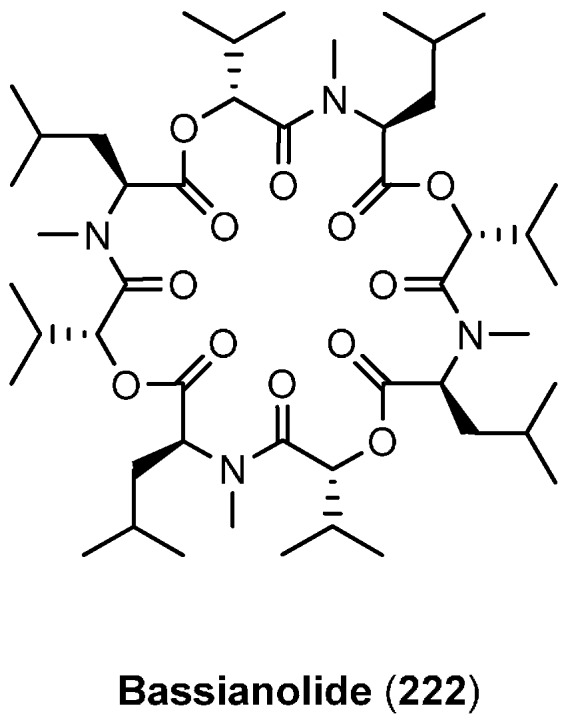
Structure of bassianolide (**222**).

Bassianolide (**222**, Figure 23) was isolated from *Beauveria bassiana*, *Lecanicilium* sp. (formerly *Verticillium lecanii*) and from the wood-decaying fungus *Xylaria* sp. BCC1067 [93]. Bassianolide belongs to the regular 24-membered cyclooctadepsipeptides and consists of four *N*-methyl-l-leucines and four d-hydroxyisovaleric acids. Manifold biological activities have been reported for bassianolide, among those insecticidal activity and cytotoxicity against different cancer cell lines. An impressive anthelmintic activity was observed with a worm (*Ascaridia galli* in chicken) reduction of ˃90% at a dosage of ≥10 mg/kg [94,95].

Bassianolide (**222**) has been synthesized in solution with standard methodology by successive couplings of Cbz-l-*N*-methylleucine with *t*-butyl-d-hydroxyisovalerate, protecting group removal and subsequent macrocyclization under high-dilution conditions [96].

### 7.2. Verticlide

**Figure 24:**
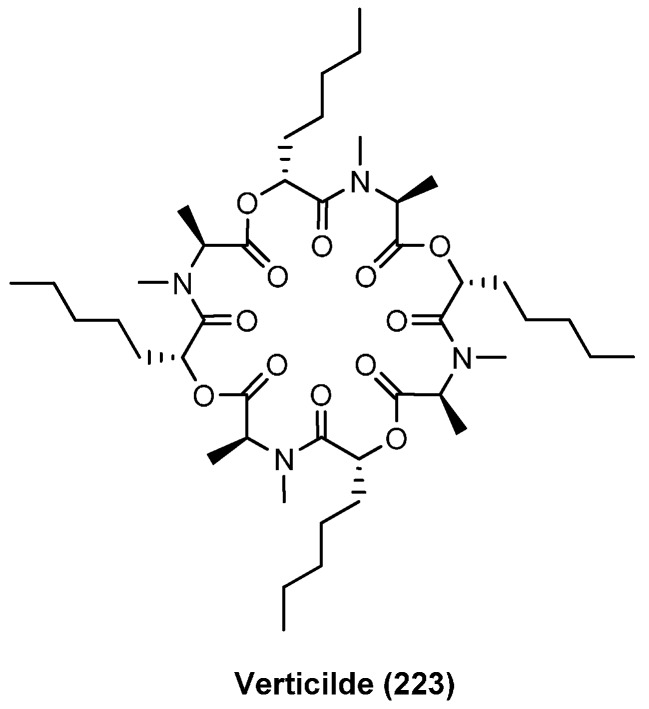
Structure of verticilide (**223**).

**Figure 25:**
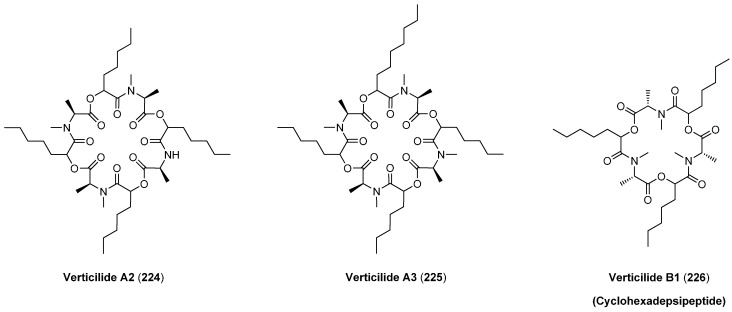
Structures of verticilide analogues **224**–**226**.

Verticilide (A1, **223**), another cyclooctadepsipeptide is produced by *Verticillium* sp. FKI-1033 (Figure 24). Similar to bassianolide verticilide is highly symmetrical and composed of four *N*-methyl-l-alanines and four d-hydroxyheptanoic acids. Verticilide (**223**) has been shown to bind selectively to the insect ryanodine receptor, a major target for modern insecticides, in the low micromolar range (IC_50_ 4.2 µM) [97]. Ryanodine receptors belong to a group of calcium channels found in skeletal muscle, smooth muscle and heart muscle cells. In contrast to mammalians, which have three types of ryanodine receptors (RyR), insects have only a single RyR. Several state of the art synthetic insecticides such as the anthranilic diamides bind to insect ryanodine receptors with nanomolar affinities. In addition to verticilide A1, three related cyclodepsipeptides, verticilides A2 (**224**), A3 (**225**) and the cyclohexadepsipeptide B1 (**226**) (Figure 25), were isolated from the fungal strain FKI-2679 by Oshiro in 2012 [98]. In addition to their ryanodine-receptor binding all verticilides inhibit efficiently cholesterol acetyltransferases ACAT1 and ACAT2 with IC_50_ values from 0.23 to 11 µM (Table 6).

**Table 6:** Inhibition of cholesterol acetyltransferases ACAT1 and ACAT2.

Verticilde	IC_50_ (µM)	
	ACAT1	ACAT2
**A1 (223)**	2.5	0.23
**A2 (224)**	4.8	0.55
**A3 (225)**	3.5	0.36
**B1 (226)**	11	1.3

**Scheme 13:**
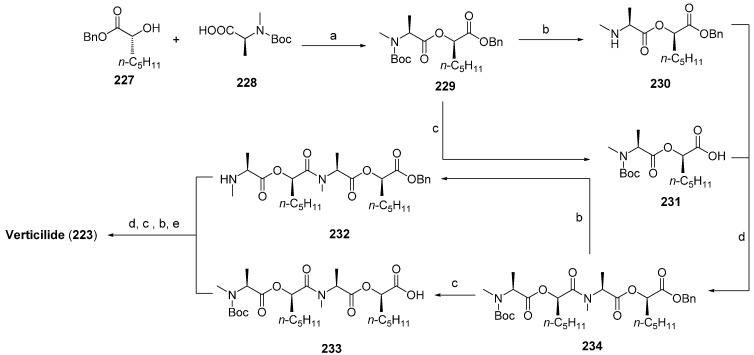
Total synthesis of verticilides (**223**).

A solution synthesis of verticilide (**223**) based on a Boc/benzyl ester protecting group strategy was published in 2006 by Omura. Including the precedent deprotection of the *N*- and *C*-termini, the macrocyclization step under high dilution conditions afforded a most remarkable 94% yield of verticilide. Overall, cyclodepsipeptide **223** was obtained in 13 steps with 66% yield (Scheme 13) [99].

**Reagents and conditions:** (a) EDCl, DMAP; (b) 4 M HCl; (c) H_2_ Pd/C; (d) PyBrop, DIPEA; (e) PyBrop, DIPEA, CH_2_Cl_2_ (0.005 M).

### 7.3. PF1022A and Emodepside

**Figure 26:**
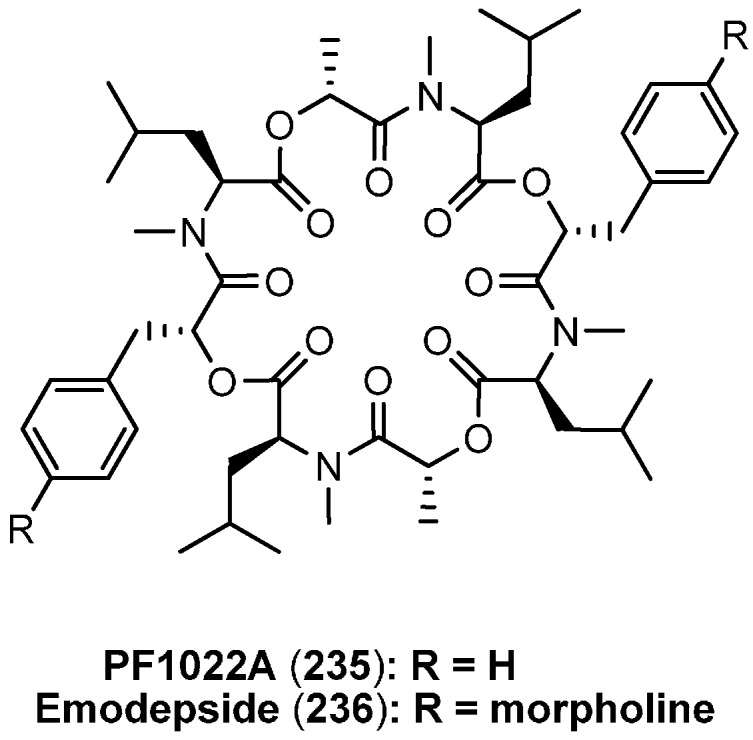
Structures of PF1022A (**235**) and emodepside (**236**).

PF1022A (**235**, Figure 26), the most prominent cyclooctadepsipeptide, was isolated in 1992 by Sasaki, who also described the potent anthelmintic activity against *Ascaridia galli* in chicken with no adverse toxic effects on the host animals [100]. PF1022A (**235**) is a metabolite of *Mycelia sterilia* (Rosselinia sp.) originally isolated from the leaves of *Camellia japonica*. PF1022A consists of four l-*N*-methylleucines, two d-phenyllactic acids and two d-lactic acids, which are linked together in a regular pattern giving the molecule a two-fold axis of symmetry. Due to its high and broad *in vitro* and *in vivo* anthelmintic activity against a multitude of nematodes, infecting humans, pets, sheep, cattle and horses, PF1022A became the most relevant lead structure in the search for novel anthelmintic drugs during the past two decades. Its unique resistance breaking mode of action, renders PF1022A superior to all standard commercial anthelmintics to which resistances have been emerged in different degrees. A semisynthetic derivative of PF1022A, emodepside (**236**, *bis-*para morpholino-PF1022A) has been introduced into the market (Profender^®^ and Procox^®^) in 2008 as a broad spectrum anthelmintic.

**Figure 27:**
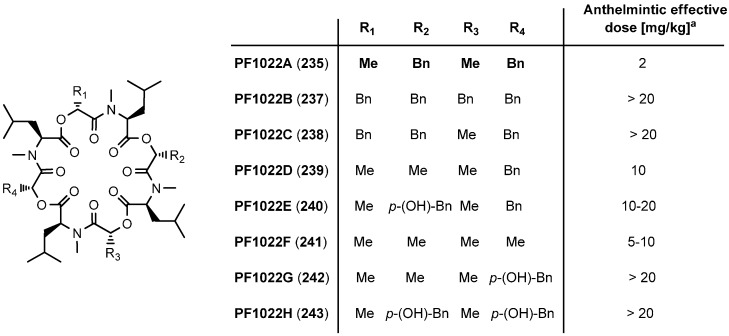
PF1022A and analogues **237**–**243** isolated from the fermentation broth.

In the fermentation broth of PF1022A (**235**), several structurally similar cyclooctadepsipeptides were found, isolated and tested for their anthelmintic activities (Figure 27) [101]. However, all homologues showed a reduced anthelmintic activity against *A. galli* in chicken.

(a) >90% worm reduction against *Ascaridia galli* in chicken.

#### 7.3.1. Syntheses of PF1022A

**Scheme 14:**
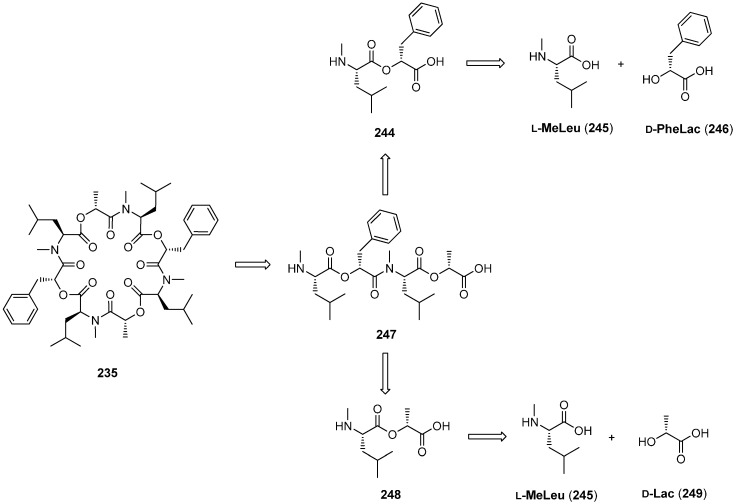
Retrosynthetic study of PF1022A (**235**).

In the past 20 years several total syntheses, both in solution and on solid-phase have been reported for PF1022A and analogues [102,103,104,105]. The *C_2_*-axis of symmetry reduces the effort to the synthesis of an appropriate substituted linear tetradepsipeptide which is subsequently dimerized and cyclized (Scheme 14).

One of the first total syntheses was described by Nelson in 1994 employing the *N*-Boc/benzylester protecting group strategy. Both amide and ester bonds of the linear depsipeptides were formed with DCC. The macrocylization reaction with BOP as coupling reagent BOP/NMM under high dilution conditions (1 mM) yielded PF1022A in a yield of 50%.

**Scheme 15:**
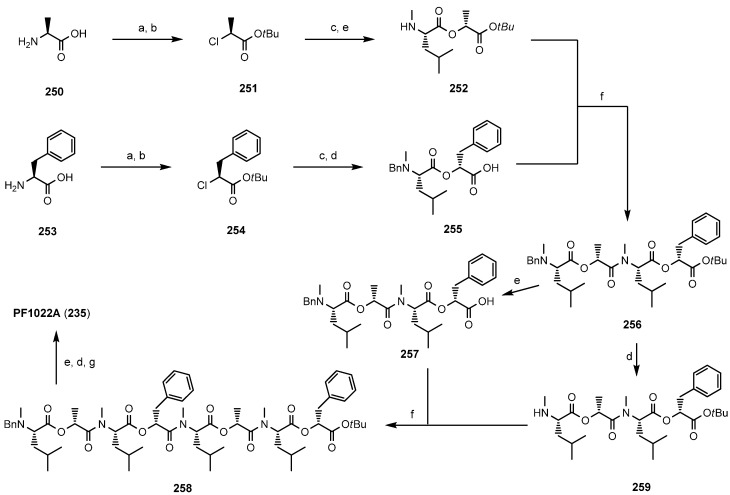
Total synthesis of PF1022A (**235**) in solution by Scherkenbeck *et al.*

A more efficient total synthesis was described by Scherkenbeck in 1995 favoring a *N*-benzyl/*tert-*butylester protecting group strategy (Scheme 15). The didepsipeptides were established with concomitant inversion of the configuration of the l-hydroxy acids introduced in the synthesis. As a consequence, only the inexpensive natural l-amino acids were needed as starting material for this total synthesis strategy. All amide bonds were established with BOP-Cl, which is known to be highly efficient in the coupling of *N*-methyl amino acids. In particular, the macrocyclization with this reagent succeeded in amazingly high yields of 85%–90% under high-dilution conditions. This remarkably high yield of an usually critical macrocyclization can be attributed to a conformer with a cis-amide bond between a Lac and a *N*-Me-Leu reside, found in the linear PF1022A precursor in solution. That *cis*-arrangement orients the *N*- and *C*-termini close together and thus facilitates the ring-closure.

**Reagents and conditions: **(a) NaNO_2_, 6 N HCl, from 0 °C to rt; (b) isobutene, conc. H_2_SO_4_, CH_2_Cl_2_, rt, autoclave, 48 h; (c) Cs_2_CO_3_, ethanol, rt, 5 h, then *N*Bn-l-Leu-O*t*Bu, DMSO, rt, 20 h; (d) Pd(OH)_2_, H_2_, ethanol, rt, 6 h; (e) HCl (gas), CH_2_Cl_2_, 0 °C, 2 h; (f) DIPEA, BOP-Cl, CH_2_Cl_2_, from 0 °C to rt, 2 h; (g) DIPEA, BOP-Cl, CH_2_Cl_2_, rt, 24 h (2 cycles).

In 1997 Lee published the first solid-phase synthesis of PF1022A (**235**) analogues, using the Kaiser-oxime resin and a Boc protecting group strategy [106]. Unfortunately, the yields of this synthesis were low and only two chain-elongation steps were performed on the resin, establishing exclusively amide-bonds. PyBrop, used for the coupling steps works especially well, like BOP-Cl, for bulky and N-alkylated amino acids. The starting tetradepsipeptide was prepared in solution as well as the didepsipeptides. As a consequence, all ester bonds were formed in solution. The Kaiser oxime is one of the rare solid-supports which allows a cyclizative cleavage under almost neutral conditions [107,108]. Thus, PF1022 analogues were obtained in about 55% yield after refluxing the deprotected linear precursor in ethyl acetate for 2 days. In the same manner Lee prepared ɛ-lactam and related analogues of PF1022A (Figure 29) [109,110].

**Scheme 16:**
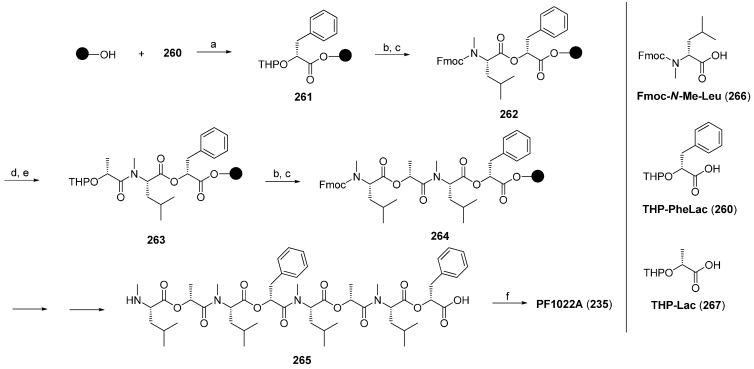
Stepwise solid-phase synthesis of PF1022A by Scherkenbeck *et al*.

In 2012 Scherkenbeck published the first real solid-phase synthesis of PF1022A based on the Wang resin and a Fmoc/THP-ether protecting group strategy [111]. That strategy allowed a stepwise coupling of *N*-methyl amino acids and hydroxy acids in an alternating sequence on solid support (Scheme 16). The Kaiser-oxime turned out unsuitable for this 17 step synthesis due to the formation of inseparable side-products which became dominant particular in the last steps. Much better results were obtained on Wang-resin with HATU as coupling reagent for amide bonds and a combination of DIC/DMAP/HOBt for the ester bonds. The overall yield obtained for the linear cyclooctadepsipeptide **265** was in the range of 13%–16%, correlating with 89% average yield for each step. The macrocyclization with BOP-Cl was accomplished in solution after removal of the protecting groups in a yield of 81%.

**Reagents and conditions:** (a) DIC, HOBt, THF, rt, 16 h; (b) *p*-TsOH, DCM/MeOH 97:3, rt, 2x1 h; (c) **266**, DIC, HOBt, DMAP, THF, rt, 16 h; (d) 25% piperidine/THF, rt, 30 min; (e) **267**, HATU, DIPEA, THF, rt, 16 h.

**Scheme 17:**
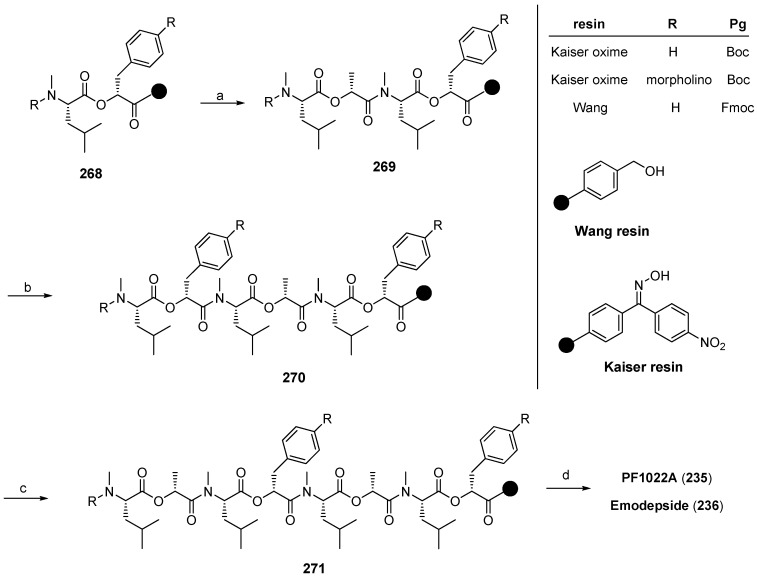
Segment solid-phase synthesis of PF1022A (**235**) and emodepside (**236**).

A segment total synthesis based on couplings of the didepsipeptides prepared in solution developed by the same group worked well on both Wang- and Kaiser-resin (Scheme 17) [112]. The advantage of this procedure is, that in the coupling steps solely amide bonds are formed which succeeds in higher yields as ester formation. Expectedly, a cyclizative cleavage strategy in a solution of DIPEA and HOAc in DCM was used to remove the final product from Kaiser oxime. The overall yield of PF1022A prepared on Kaiser-oxime was 30% and that of emodepside remarkable 45% compared to Wang resin where PF1022 (**235**) was obtained only in 25% yield including the separate solution cyclization.

#### 7.3.2. Synthesis of PF1022A-Analogues via Total Synthesis

**Figure 28:**
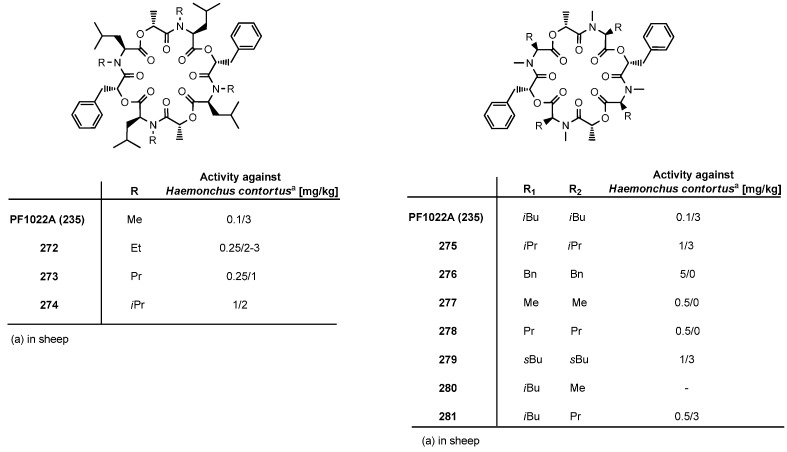
Biological activity of various PF1022A-derivatives.

In order to establish structure-activity relationships, numerous PF1022A analogues were prepared either by total synthesis or by semisynthetic modification of the natural product. The significance of the *N*-methyl groups was tested by replacing them with *N*-ethyl, *N*-propyl and *N*-isopropyl groups. While the *N*-ethyl PF1022 analogue had almost the same anthelmintic activity as the natural product, the *N*-propyl and *N*-isopropyl derivatives were clearly less active. A PF1022A analogue lacking all *N*-methyl groups was found almost inactive, too. The exchange of *N*-Me-Leu for *N*-Me-Ile, *N*-Me-Val, *N*-Me-Nva (*N*-methyl-norvaline), *N*-Me-Ala and *N*-Me-Phe also resulted in reduced anthelmintic activities (Figure 28).

**Reagents and conditions:** (a) Kaiser oxime: i. 25% TFA/DCM, rt, 30 min; ii. Boc-MeLeu-Lac-OH, HATU, DIPEA, DMF, rt, 16 h; iii. 25% TFA/DCM, rt, 30 min; Wang resin: i. 25% piperidine/THF, rt, 30 min; ii. Fmoc-MeLeu-Lac-OH, HATU, DIPEA, THF, rt, 16 h; iii. 25% piperidine/THF, rt, 30 min; (b) Kaiser oxime: i. Boc-MeLeu-PhLac-OH or Boc-MeLeu-morphPhLac-OH, HATU, DIPEA, DMF, rt, 16 h; ii. 25% TFA/DCM, rt, 30 min; Wang resin: i. Fmoc-MeLeu-PhLac-OH, HATU, DIPEA, THF, rt, 16 h; ii. 25% piperidine/THF rt, 30 min; (c) Kaiser oxime: i. Boc-MeLeu-Lac-OH, HATU, DIPEA, DMF, rt, 16 h; ii. 25% TFA/DCM, rt, 30 min; Wang resin: i. Fmoc-MeLeu-Lac-OH, HATU, DIPEA, THF, rt, 16 h; ii. 25% piperidine/THF, rt, 30 min; (d) Kaiser oxime: DIPEA, AcOH, DCM, rt, 16 h; Wang resin: i. 50% TFA/DCM, rt, 1 h: ii. BOP-Cl, DIEA, rt, 48 h.

Based on the hypothesis, that only one half of the C_2_-symmetric PF1022A cycle is necessary for biological activity, unsymmetric PF1022A derivatives were synthesized by replacing the *N*-methyl-leucines in one half of the molecule by *N*-methyl-alanine and *N*-methyl-norvaline residues [113]*.* However, also those derivatives were found considerably less anthelmintically active. The crucial role of the hydroxy acids becomes already evident from a comparison of PF1022A (**235**) with PF1022B (**237**) and PF1022C (**238**) which show a reduced anthelmintic activity against *H. contortus* at least by a factor of 10 (Figure 27). Anyway, the position of choice for structural modifications turned out to be the phenyllactic acid residues as exemplified by emodepside (**236**).

**Figure 29:**
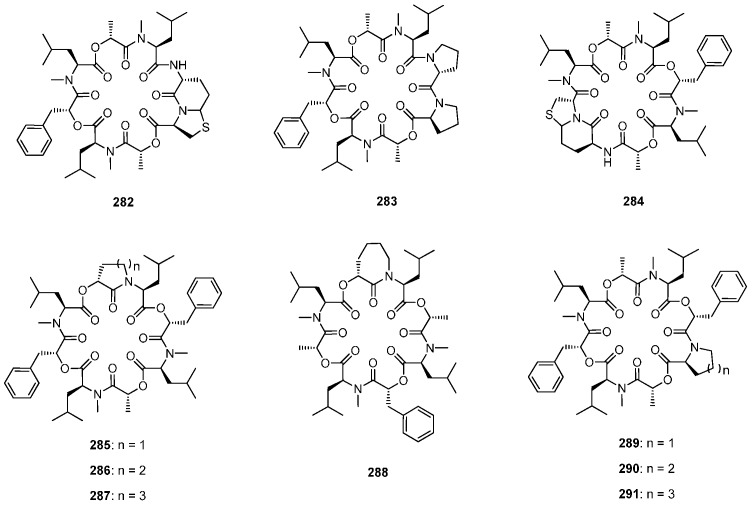
Conformationally restricted analogues of PF1022A (**235**).

Another line of research besides total syntheses of PF1022A analogues, derivatization of the natural product and backbone modifications was addressed to the question of conformational flexibility of the cyclooctadepsipeptide macrocycle. In solution PF1022A exists as a mixture of two conformers, one of which contains a single *cis*-amide bond between a *N*-Me-Leu and a Lac residue as part of a β-turn [114]. In order to elucidate the role of the β-turn, Scherkenbeck replaced a *N*-Me-Leu-Lac moiety by the more rigid d-Pro-l-Pro dipeptide and the bicyclic “Nagai-Sato” (BTD) bicyclic β-turn mimic (Figure 29) [115]. Analogous, conformationally restricted analogues **285**–**287** and **289**–**291** were reported by Dutton [116]. Cyclodepsipeptides **282** and **284** gave full control of *H. contortus* at a dose of 0.1 mg/kg in sheep while the d-Pro-l-Pro analogue **283** was found almost inactive (Figure 29). These results indicate that by an appropriate choice of the β-turn mimetic at least partially non-peptidic PF1022A analogues may be feasible with improved metabolic stability.

**Scheme 18:**
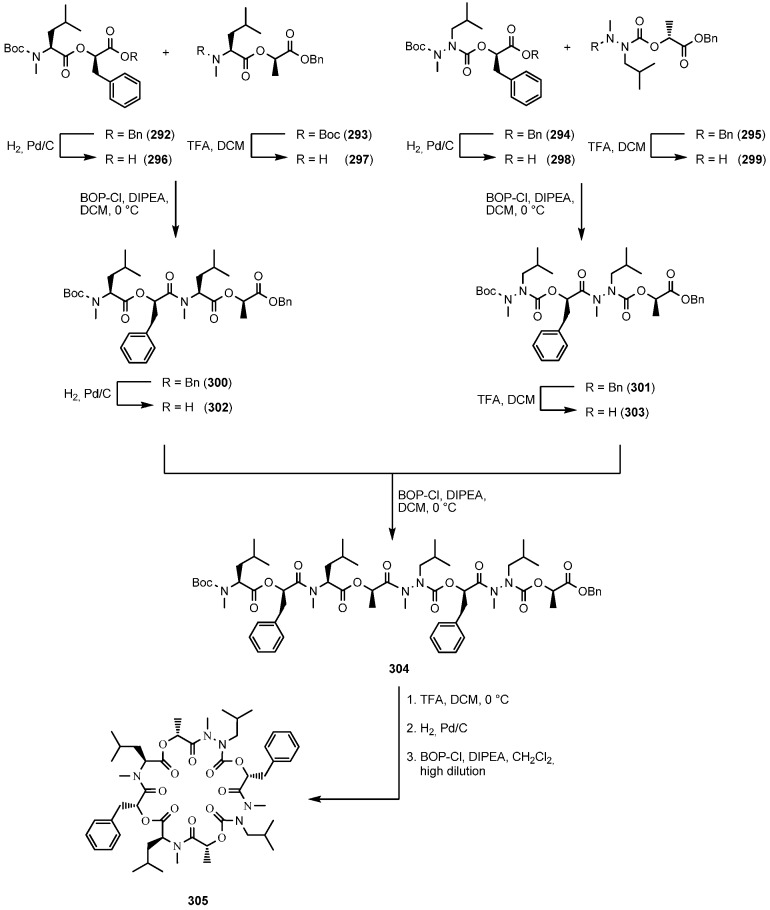
Synthesis of aza-PF1022A (**305**).

Research published by Scherkenbeck on the novel class of azadepsipeptides goes in the opposite direction and aims at imposing more flexibility on the cyclooctadepsipeptide framework [117]. In a lengthy synthesis (Scheme 18) the azadepsipeptide analogue **305** was prepared and tested for its anthelmintic activity. Again, the anthelmintic activity was reduced considerably. However, the reason for that remains speculative because two parameters have been changed at the same time. The α-carbon of leucine was replaced by a nitrogen atom and at the same time the chirality at that position was destroyed.

#### 7.3.3. PF1022A Analogues by Direct Derivatization of the Natural Product

**Scheme 19:**
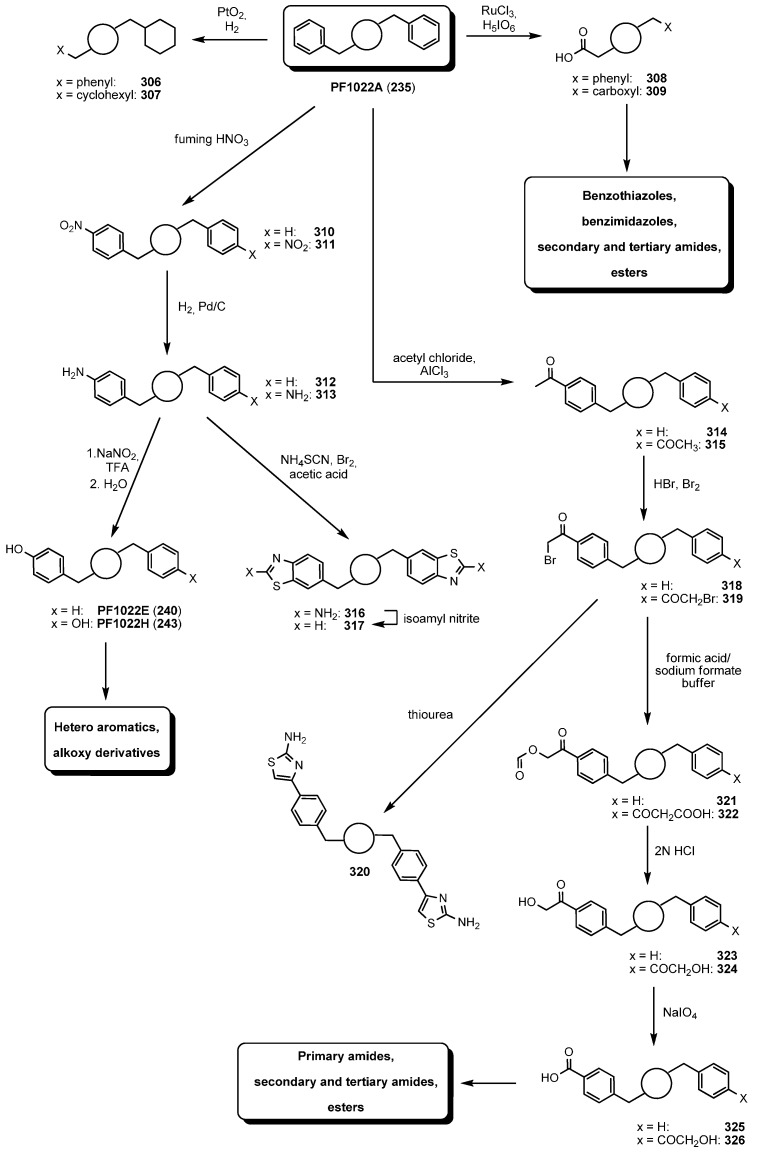
Derivatization of PF1022A (**235**).

PF1022A has a remarkable stability against strongly acidic conditions. Therefore, classical aromatics chemistry such as nitrations, sulfonylations, halogenations and Friedel-Crafts acylations have been used to prepare a multitude of PF1022A analogues by modification of the natural product (Scheme 19) [118]. On the contrary, Palladium catalyzed coupling reactions do not work at all, probably due to complexation of the Palladium in the PF1022A macrocycle.

A catalytic oxidation of the benzene rings of PF1022A (**235**) with RuCl_3_ provides the *mono*- or *bis*-carboxylic acids **308** and **309**, which represent valuable starting materials for the synthesis of diverse heteroaromatic systems such as benzothiazoles and benzimidazoles [119]. The *tert*-butyl and related alkyl ethers of PF1022H, a side-product from fermentation, showed very promising anthelmintic profiles. An alternative to the minute amounts of PF1022H accessible from the fermentation broth is based on the *bis-*anilino PF1022A (**313)** which is available by nitration of PF1022A with fumic nitric acid and subsequent hydrogenation. Formation of the diazonium salt and aqueous work-up immediately provided semisynthetic PF1022H (**243**) in excellent yields (80%). In addition, the *bis-*anilino PF1022A (**313)** has been used for the preparation of urethanes, carbamates and heterocylic analogues. Emodepside (**236**) was prepared from the *bis-*anilino derivative **313** by alkylation with the *bis-*tosylates in high yields.

Friedel-Crafts acylation of PF1022A with acetyl chloride followed by bromination provides the α-bromo ketones **318** and **319** as starting materials for Hantzsch syntheses of pyrroles, thiazoles and additional heteroaromatics. By systematic modification of the phenyl rings or replacement by other heteroaromatics several highly anthelmintic PF1022A derivatives were identified over the past decade.

**Figure 30:**
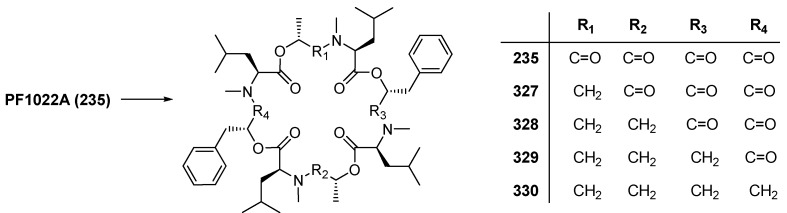
Backbone modification of PF1022A by reduction using BH_3_·THF.

Further modifications were directed to the PF1022A backbone including partial reductions of the amide groups and chemoselective transformations of the carbonyl groups to oximes and thioamides [120]. With increasing amounts of BH_3_ THF and reaction times it is possible to reduce two, three or all amide bonds. Due to a lower steric hindrance the MeLeu-Lac amide bonds are reduced first, followed by the MeLeu-PheLac bonds (Figure 30). An anthelmintic screening against *H. contortutus* in sheep demonstrated the *mono*- and *bis*-reduction products **327** and **328** highly active, while the *tri*- and *tetra*-reduced products **329** and **330** retained only weak anthelmintic activity.

**Scheme 20:**
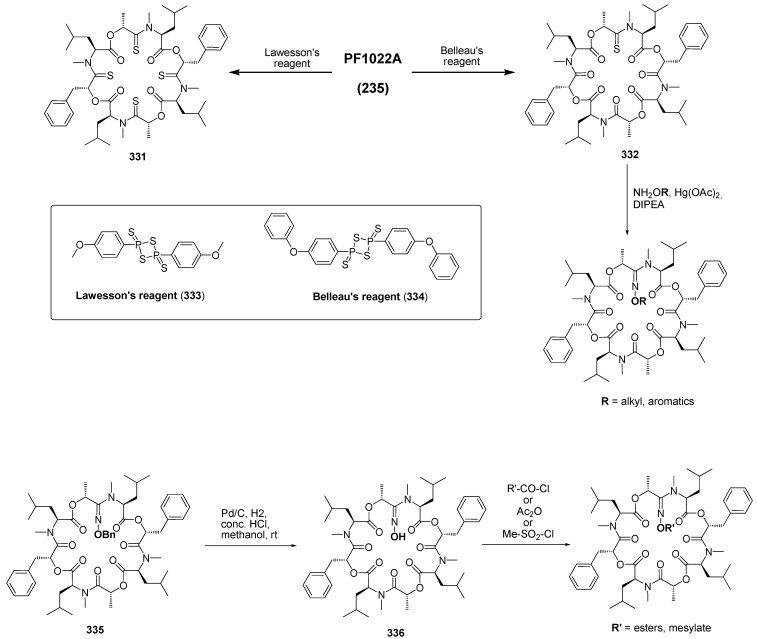
Thionation of PF1022A and conversion to amidoximes.

Additional backbone-modified PF1022A derivatives were prepared by chemoselective thionation of the amide carbonyl groups [121]. With Belleau’s reagent (**334**, 0.5 molar equiv. in tetrahydrofuran) a *mono*-thionated product was obtained, with an excess of Lawesson’s reagent (**333**) all amide carbonyl groups were transformed into thioamides, respectively (Scheme 20). Subsequent reaction of the thioamides with substituted hydroxylamines in the presence of Hg(OAc)_2_ afforded the corresponding amidoximes some of which showing better anthelmintic activity than PF1022A [122].

#### 7.3.4. Biosynthesis of PF1022A

**Figure 31:**
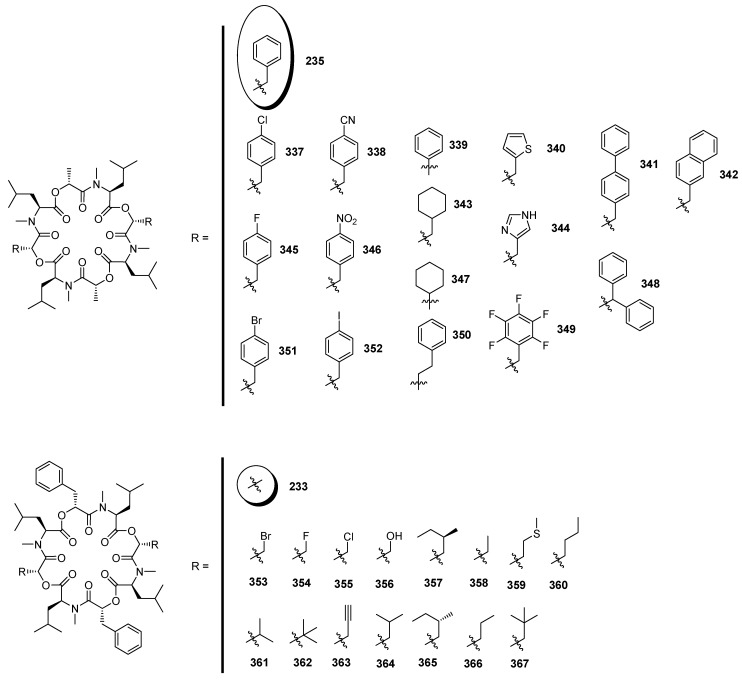
PF1022A derivatives obtained by *in vitro* synthesis.

PF1022A (**235**) is biosynthesized by the nonribosomal peptide synthetase PFSYN (350 kDa) following the usual thiotemplate mechanism [123]. The substrate tolerance of PFSYN for hydroxy acids was explored comprehensively by Süssmuth (Figure 31) [124]. PFSYN was shown to accept a broad variety of aromatic, heteroaromatic and aliphatic residues which were successfully incorporated into the cyclooctadepsipeptide framework allowing the synthesis of a broad spectrum of PF1022A derivatives. In contrast to the α-hydroxy activating domain of enniatin synthetase (ESYN), PFSYN is capable of activating both d-lactates and d-phenyllactates. In particular mentionable with respect to more advanced analogues is the acceptance of hydroxy acids with *para*-halogenated phenyl rings and propargyl side-chains.

#### 7.3.5. Mode of Action of PF1022A and Emodepside

PF1022A (**235**) and its semi-synthetic commercial derivative emodepside (**236**) show high activity against several parasitic gastrointestinal nematodes in combination with low toxicity to mammals. Most important, **235** and **236** are fully effective against levamisol-, benzimidazol- and ivermectin-resistant nematodes in sheep and cattle. This fact strongly supports the hypothesis of a novel and unique mode of action for PF1022A and related cyclooctadepsipeptides [125]. The application of PF1022A or emodepside induces the release of inhibitoric, postsynaptic acting neurotransmitters, resulting in the paralysis of pharyngeal muscles and the somatic musculature which finally causes the death of the nematode. The elucidation of the molecular mode of action of PF1022A was an odyssey for almost 20 years. Beginning with the ionophoric properties as the toxic principle of PF1022A and related cyclodepsipeptides, followed by the suggestion of a direct interaction with the GABA receptor, mode of action studies then postulated the HC110-R receptor, related to the mammalian G-protein coupled receptor latrophilin, to be the molecular target of PF1022A. Latrophilin is the known receptor of latrotoxin, the poison of the black widow spider. Most recent studies propose the SLO-1 potassium channel as the real target eventually together with HC110-R [126].

## 8. Cyclononadepsipeptides

### 8.1. BZR-cotoxins I-IV

**Figure 32:**
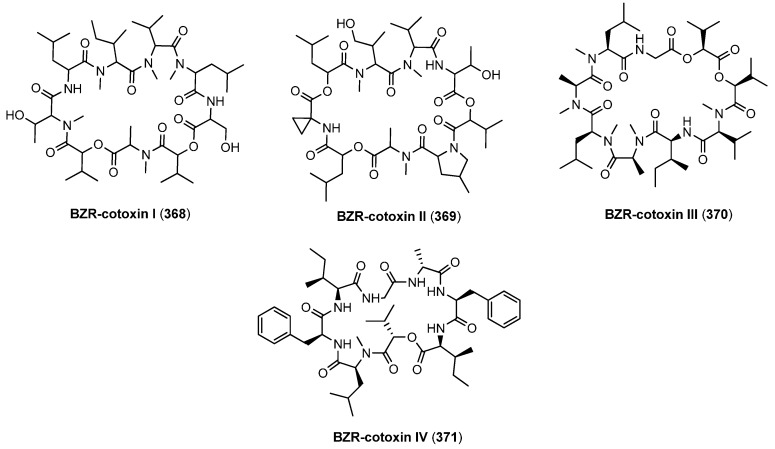
Structures of BZR-cotoxin I-IV.

BZR-cotoxins (Figure 32) were isolated from the plant pathogenic fungus *Bipolaris zeicola*. The BZR-cotoxin complex consists of three cyclononadepsipeptides (BRZ-cotoxin I-III, (**368**–**370**) and the cyclooctadepsipeptide BZR-cotoxin IV (**371**). Among those four cyclodepsipeptides BZR-cotoxin IV was considered the most important one [127,128,129,130].

### 8.2. Aureobasidins

**Figure 33:**
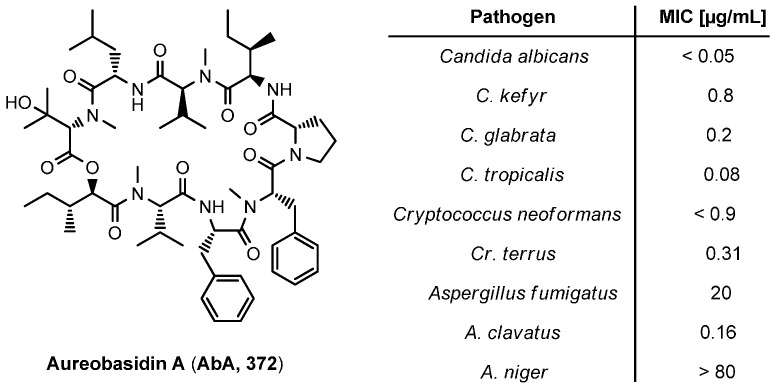
Structure and activity of aureobasidin A (**372**).

Aureobasidins, isolated from the black yeast *Aureobasidium pullulans* R106, show high antifungal activities (Figure 33). To date over 30 aureobasidin analogues were characterized, all of which are composed of one hydroxy and eight amino acids [131,132]. A central step in the total synthesis of aureobasidin A (**372**) by Kuromoe is the PyBrop mediated macrocycle formation between l-*allo*-isoleucine (l-*a*Ile) and l-Pro [133]. Another total synthesis, described by Cooper and coworkers uses fragment couplings and a cyclization between the residues l-Phe and l-Val [134].

**Scheme 21:**
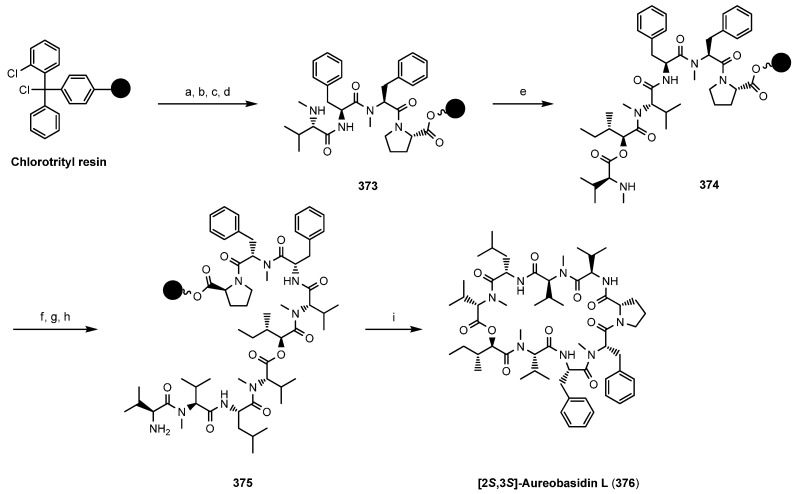
Total synthesis of aureobasidin derivative **376** on solid-phase.

Recently Maharani *et al.*, described a total synthesis of an synthetic analogue of (**372**), [2*S*,3*S*]-aureobasidin L (**376**), on chlorotrityl resin [135]. The critical macrocyclization was accomplished by formation of an amide bond between Val (*N*-terminus) and Pro (*C*-terminus) (Scheme 21).

Reagents and conditions: (a) 1. Fmoc-l-Pro, CH_2_Cl_2_, DIPEA; 2. MeOH; 3. piperidine in DMF; (b) 1. Fmoc-l-*N*-MePhe, HBTU/HOBt, HATU/HOAt, or BTC (triphosgene)/collidine, DIPEA, CH_2_Cl_2_/DMF; 2. piperidine in DMF; (c) 1. Fmoc-l-Phe, HATU/HOAt, DIPEA, CH_2_Cl_2_/DMF; 2. piperidine in DMF; (d) Fmoc-l-*N*-MeVal, HBTU/HOBt or HATU/HOAt, DIPEA, CH_2_Cl_2_/DMF; 2. piperidine in DMF; (e) 1. Fmoc-l-*N*-MeVal-l-Hmp-OH, BTC/collidine, DIPEA, THF; 2. 20% piperidine in DMF (two cycles) or piperidine/DBU/DMF (two cycles); (f) 1. Fmoc-l-Leu, BTC/collidine, DIPEA, THF then HATU/HOAt, DIPEA, CH_2_Cl_2_/DMF; 2. 20% piperidine in DMF (two cycles) or piperidine/DBU/DMF, 20 min; (g) 1. Fmoc-l-*N*-MeVal, BTC/collidine, DIPEA, THF; 2. piperidine/DBU/DMF, 20 min; (h) 1. Fmoc-l-Val, BTC/collidine, DIPEA, THF then HATU/HOAt, DIPEA, CH_2_Cl_2_/DMF; 2. piperidine/DBU/DMF, 20 min; (i) 1. TFA/CH_2_Cl_2_, 15 min; 2. HATU, DIPEA, CH_2_Cl_2_.

## 9. Cyclodecadepsipeptides

### 9.1. Clavariopsin A and B

**Figure 34:**
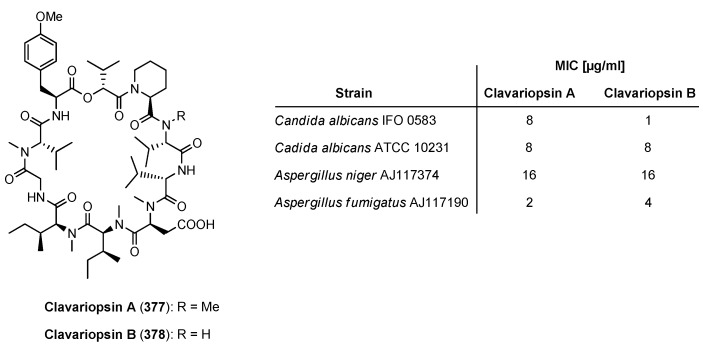
Structures of clavariopsins A and B and their antifungal activity.

The antifungal active Clavariopsins A (**377**) and B (**378**) (Figure 34) were isolated from the culture broth of the aquatic hyphomycetes *Clavariopsis aquatic*. These decacyclodepsipeptides induce a swelling of fungal hyphae by inhibition fungal cell wall biosynthesis [136,137].

## 10. Summary

This review gives an overview of natural depsipeptides with ring-sizes between 12 and 30 atoms and composed exclusively of *α*-amino acids and at least one α-hydroxy acid. Most of these macrocyclic depsipeptides show impressive biological activities in the field of crop protection, animal health or medicine. The high incidence of biological activity in this family of natural products can be attributed to the cyclic nature which improves the metabolic stability and the occurrence of α-hydroxy acids which mimic α-amino acids. The biosynthesis of cyclodepsipeptides is accomplished nonribosomally by cyclodepsipeptide synthetases, which comprise large multifunctional proteins. The cyclodepsipeptide synthases show a relatively broad tolerance for α-hydroxy acids and have been applied for *in vitro* syntheses of new cyclodepsipeptides, simply by adding synthetic α-hydroxy acids to those enzymes. Numerous cyclodepsipeptides such as the AM-toxins, the kutznerides and the monamycins contain unusual nonribosomal residues which present formidable challenges also for the total synthesis of these compounds. Highly efficient syntheses both in solution and on solid-phase have been developed for enniatins and in particular for the cyclooctadepsipeptide PF1022A. The most critical step in almost all syntheses is the final macrocyclization which due to entropic reasons needs high-dilution conditions and often provides only moderate yields of the cyclization product. Chemical syntheses, *in vitro* syntheses with the cyclodepsipeptide synthetases and feeding experiments using the cyclodepsipeptide producing strains have been employed for the efficient production of analogues of the natural cyclodepsipeptides.

Presently, the most successful story is the development of the semi-synthetic PF1022A derivative emodepside as a commercial anthelmintic. Intelligent combinations of classical total syntheses, chemical derivatizations and (bio)combinatorial approaches will provide additional commercial cyclodepsipeptides in the fields of medicine and plant protection in the near future, underlining the importance of this particular class of natural compounds.
